# Exceptional Changes in Skeletal Anatomy under Domestication: The Case of Brachycephaly

**DOI:** 10.1093/iob/obab023

**Published:** 2021-08-16

**Authors:** M Geiger, J J Schoenebeck, R A Schneider, M J Schmidt, M S Fischer, M R Sánchez-Villagra

**Affiliations:** Paleontological Institute and Museum, University of Zurich, Karl-Schmid-Str. 4, 8006 Zurich, Switzerland; Roslin Institute and Royal (Dick) School of Veterinary Studies, University of Edinburgh, Easter Bush Campus, Midlothian EH25 9RG, UK; Department of Orthopaedic Surgery, University of California at San Francisco, 513 Parnassus Avenue, S-1164, San Francisco, CA 94143-0514, USA; Clinic for Small Animals—Neurosurgery, Neuroradiology and Clinical Neurology, Justus Liebig University Giessen, Frankfurter Str. 114, 35392 Giessen, Germany; Institute of Zoology and Evolutionary Research, Friedrich-Schiller University Jena, Erbertstr. 1, 07743 Jena, Germany; Paleontological Institute and Museum, University of Zurich, Karl-Schmid-Str. 4, 8006 Zurich, Switzerland

## Abstract

“Brachycephaly” is generally considered a phenotype in which the facial part of the head is pronouncedly shortened. While brachycephaly is characteristic for some domestic varieties and breeds (e.g., Bulldog, Persian cat, Niata cattle, Anglo-Nubian goat, Middle White pig), this phenotype can also be considered pathological. Despite the superficially similar appearance of “brachycephaly” in such varieties and breeds, closer examination reveals that “brachycephaly” includes a variety of different cranial modifications with likely different genetic and developmental underpinnings and related with specific breed histories. We review the various definitions and characteristics associated with brachycephaly in different domesticated species. We discern different types of brachycephaly (“bulldog-type,” “katantognathic,” and “allometric” brachycephaly) and discuss morphological conditions related to brachycephaly, including diseases (e.g., brachycephalic airway obstructive syndrome). Further, we examine the complex underlying genetic and developmental processes and the culturally and developmentally related reasons why brachycephalic varieties may or may not be prevalent in certain domesticated species. Knowledge on patterns and mechanisms associated with brachycephaly is relevant for domestication research, veterinary and human medicine, as well as evolutionary biology, and highlights the profound influence of artificial selection by humans on animal morphology, evolution, and welfare.

## Zusammenfassung

Als “Brachycephalie” wird im Allgemeinen ein Phänotyp bezeichnet, der sich durch einen stark verkürzten Gesichtsschädel auszeichnet. Obwohl Brachycephalie für manche Haus- und Nutztiere ein rassenspezifisches Merkmal ist (z.B. bei der Bulldogge, der Perserkatze, dem Niata-Rind, der Anglo-Nubischen Ziege und dem Middle White-Schwein), kann dieser Phänotyp auch pathologisch sein. Trotz der oberflächlichen Ähnlichkeit des “brachycephalen” Phänotyps in diesen Varietäten und Rassen, zeigt ein detaillierter Vergleich ihrer Schädelmorphologie, dass “Brachycephalie” eine Vielzahl unterschiedlicher Schädelveränderungen umfasst, welche wahrscheinlich unterschiedliche genetische und entwicklungsbiologische Grundlagen haben. In dieser Übersichtsarbeit schaffen wir einen Überblick über die verschiedenen Definitionen und Charakteristika, welche mit Brachycephalie in den verschiedenen domestizierten Formen assoziiert sind. Dabei unterscheiden wir zwischen verschiedenen Brachycephalie-Typen («Bulldoggen-Typus», «Katantognather Typus» und «Allometrischer Typus») und diskutieren morphologische Besonderheiten—inklusive Pathologien—welche mit der Brachycephalie in Verbindung stehen (z.B. das Brachycephale Syndrom). Wir diskutieren weiterhin die bisher bekannten, komplexen genetischen und entwicklungsbiologischen Prozesse die zu Brachycephalie führen können, sowie kulturelle und entwicklungsbiologische Gründe, weshalb Brachycephalie in gewissen Arten auftritt, während in anderen Arten keine solchen Phänotypen bekannt sind. Das Wissen um die Muster und Mechanismen, welche zu Brachycephalie führen, sind relevant für die Domestikationsforschung, die Veterinär- und Humanmedizin, sowie für die Evolutionsbiologie und betonen den tiefgreifenden Einfluss von künstlicher Selektion auf die Morphologie, die Evolution und das Tierwohl unserer Nutz- und Haustiere.

## Resumen

La braquicefalia generalmente se considera un fenotipo en el que el cráneo, específicamente el hocico, es notablemente acortado. Mientras que la braquicefalia es característica de algunas variedades domésticas y razas (p.e. Bulldog, gato persa, vaca ñata, cabra anglo nubiana, cerdo Middle White), también se puede interpretar como un fenotipo patológico. A pesar de que la braquicefalia tiene una apariencia semejante, por lo menos superficial, en estas variedades y razas, al examinarla más en detalle se descubre que la “braquicefalia” incluye una variedad de diferentes modificaciones del cráneo que probablemente tienen diferentes subyacentes genéticos y de desarrollo y que están relacionados con la historia de la raza. Revisamos las diferentes definiciones y propiedades relacionadas con la braquicefalia en varias especies domésticas. Describimos diferentes tipos de braquicefalia (tipo bulldog, “katantognático” y braquicefalia alométrica) y analizamos condiciones morfológicas relacionadas con la braquicefalia incluyendo enfermedades (p.e. síndrome obstructivo respiratorio). Además, examinamos los complejos procesos genéticos y de desarrollo subyacentes y los motivos culturales y de desarrollo por las que variedades braquicéfalas pueden ser más o menos prevalentes en ciertas especies domésticas. El conocimiento de patrones y mecanismos asociados a la braquicefalia son relevantes para la investigación sobre la domesticación, medicina veterinaria y humana, así como para la biología evolutiva y destaca la profunda influencia de la selección artificial sobre la morfología y bienestar de los animales y su evolución.

## Introduction

The domestication process in animals is generally associated with a shortening of the snout relative to the cranium (Zeuner et al. 1963; Mason 1984; Herre and Röhrs 1990; Clutton-Brock 1999; Trut, Plyusnina and Oskina 2004; Geiger, Sánchez-Villagra and Lindholm 2018). This has been hypothesized to be causally related to neural crest-driven effects of the selection for tameness (Wilkins, Wrangham and Fitch 2014), although this has been debated (Sánchez-Villagra, Geiger and Schneider 2016; Lord et al. 2019; Kistner et al. 2021). This and other comparatively subtle alterations of skull shape associated with the domestication process per se (e.g., Albarella, Dobney and Rowley-Conwy 2006) are fundamentally different from the more pronounced forms of shortened and sometimes tilted faces that are associated with the formation of particular varieties and breeds (e.g., Herre and Röhrs 1990; Van Grouw 2018). Well-known examples include Bulldogs, Pugs, and Persian cats. Such breeds are generally termed “brachycephalic.”

Brachycephaly has been the subject of significant research, including morphological characterizations and definitions (e.g., Herre and Röhrs 1990), the investigation of inheritance patterns (e.g., Stockard 1941), the study of genetic underpinnings (e.g., Fondon and Garner 2004; Bannasch et al. 2010; Bertolini et al. 2016; Marchant et al. 2017), implications for health and animal welfare (e.g., Schlueter et al. 2009; Schoenebeck et al. 2012; Packer, Hendricks and Burn 2015a; Packer et al. 2015b; Farnworth et al. 2016; Schmidt et al. 2017), and the analogy to similar human diseases (e.g., Rusbridge 2005; Lyons et al. 2016; Marchant et al. 2017). These studies have been mostly concerned with domestic dogs and cats. However, greatly shortened snouts occur also in domestic pigs, cattle, goats, and rabbits, as well as in pigeons and chickens among birds (e.g., Herre and Röhrs 1990; Clutton-Brock 1999; Veitschegger et al. 2018; Diogo et al. 2019).

The study of brachycephaly is relevant in a broad evolutionary context (Usui and Tokita 2018). For example, the peculiar skull shape of some domestic pigeon breeds, notably “brachycephalic” ones, appears to resemble that of some wild bird species (Young et al. 2017). This suggests that similar developmental processes may shape wild as well as domestic species (Young et al. 2017). Two further examples among wild species with morphology related to brachycephaly are some groups of bats (Chiroptera), which exhibit a marked anteroposteriorly flattened and dorsoventrally flexed snout (e.g., Mormoopidae) (Arbour, Curtis and Santana 2019), and among primates orangutans (*Pongo*), which exhibit a relatively short and upward tilted facial region (Selenka 1898; Shea 1985). However, these examples of “adaptive” brachycephaly are probably the result of evolutionary processes leading to increased functional efficiency of the involved oronasal structures, e.g., in bats (Arbour et al. 2019), whereas at least some cases of (extreme) brachycephaly in domestication—as well as similar phenotypes due to pathology in humans—may be associated with decreased functionality and even pathological conditions (e.g., Koch et al. 2003; note that adaptation might still play a role in brachycephaly in domestication).

The aim of this review is to provide an overview of brachycephaly in domestication in an evolutionary developmental and phylogenetic perspective. For this, we synthesize existing and new knowledge on brachycephaly across varieties/breeds of different domesticated species, with a focus on mammals. Further, we review findings on possible genetic and developmental as well as selective causes and constraints influencing the evolution of this phenotype.

## Nomenclature and definitions

Brachycephaly has been described to occur in many domesticated species (e.g., Herre and Röhrs 1990). The term “brachycephaly” originates from anthropology, where it is used to describe the shape of the cranial vault in dorsal view, characterized by length and breadth measurements (Retzius 1850; Rosenberg 1965; Lüps 1974). However, in nonhuman mammals, “brachycephaly” involves also the facial part of the skull, i.e., the anterior part of the fetal chondrocranium. Despite the widespread use of the term, there is no universal definition of what constitutes brachycephaly. The reason for this is the challenge of developing a definition that applies to many morphologically different species, as well as the continuous nature and variability of the condition. Relative snout length is a continuous characteristic and the “threshold of brachycephaly” is therefore arbitrary. In other words, should a short snout relative to the wild form always be considered brachycephalic? If not, how pronounced do the changes have to be in order to be considered brachycephalic? That this is not only an “academic” question has become evident in 2019 when the Dutch Minister for Agriculture, Nature and Food Quality has published a letter on animal welfare that forbids breeding with brachycephalic dogs (Schouters 2019; Van Hagen 2019). For such regulations to be implementable, it has to be clear what brachycephaly actually comprises. Since there are inconsistencies in the literature concerning the terminology associated or synonymized with brachycephaly (e.g., Rosenberg 1965; Lüps 1974; Harvey 1985), we describe our usage of terms in Table 1.

**Table 1:** Terms and their definitions as used in this review

Terms	Definitions	References
Airorhynchy	Dorsal rotation/upward tilting of the palate relative to the cranial base. This condition is sometimes synonymized to brachycephaly. The opposite condition, with a ventral rotation/downward tilting of the palate relative to cranial base is usually termed “klinorhynchy.”	(Rosenberg 1965; Nussbaumer 1982; Koch et al. 2003)
Allometric changes	Changes of biological variables, e.g., shape of an organ/structure such as the skull, correlated with changes of the size of the same organ/structure or overall body size. A linear scaling relationship is given as: log(*y*) = log(*a*) + b*log(*x*), where *a* is the slope and *b* the intercept. The biological variable in question (*y*) can scale with (body) size (*x*) in three different ways: isometry, no change of shape with size increase (*a* = 1); negative allometry, change of shape with size is less than isometric (*a* < 1); positive allometry, change of shape with size is more than isometric (*a* > 1). Please note that while this formula follows the traditional school (changes in relative size of traits), the definition used here and in the remainder of this review concerns the more derived but interrelated variation of shape with size.	(Huxley 1932; Gould 1966; Klingenberg 2016)
Brachycephaly	Short and wide head. Here, we mainly focus on the facial length, as the cranial width does not seem to be equally affected across species. The opposite condition is usually termed “dolichocephaly,” meaning long (or narrow) head.	(Ellenberger and Baum 1891; Evans 1993)
Brachygnathia superior = (mandibular) prognathism = undershot jaw	Short upper jaw (maxilla) and “normal”-sized lower jaw (mandible). This condition is sometimes synonymized to brachycephaly. The opposite condition is usually termed “overshot jaw,” which is characterized by a short lower jaw and a “normal”-sized upper jaw.	(Harvey 1985; Böhmer 2003; Johnston 2006)
Katantognathy	Ventral rotation/downward tilting of the premaxilla relative to the palate.	(Selenka 1898; Nussbaumer 1982)
Roman nose	Convex profile of the nose	(Porter 1996)
Simognathy	Dorsal rotation/upward tilting of the premaxilla relative to the palate	(Selenka 1898; Nussbaumer 1982)

## Brachycephaly in domestic species

**Fig. 1:**
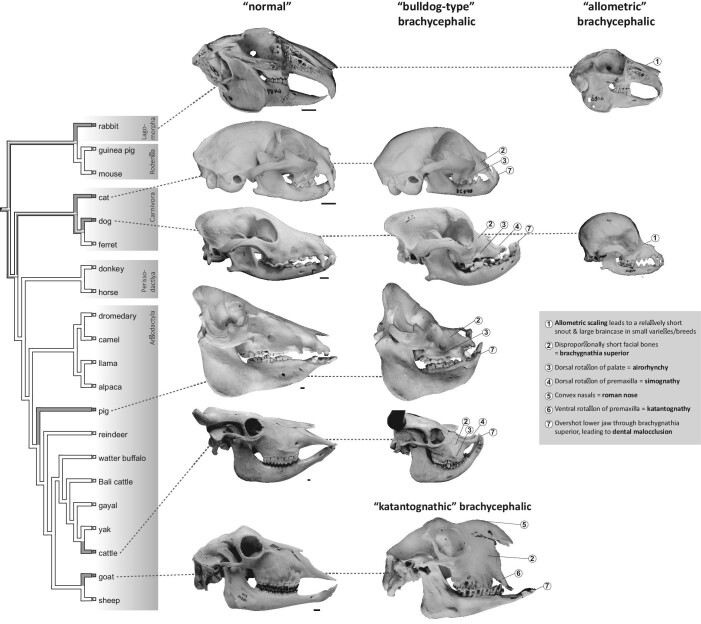
Summary of brachycephalic varieties in domestic mammal species. Cladogram (branches contain no information on divergence times) shows ancient mammal domesticates (domesticated >500 YBP, see text; tree topology is according to Meredith et al. 2011 and Agnarsson and May-Collado 2008). Gray branches indicate species with at least one variety/breed where a brachycephalic phenotype is considered to occur relatively consistently or is breed defining and not just occurring occasionally, e.g., as a pathology (see text and Table 2). Skulls categorized as “normal” (left column) represent the non-brachycephalic condition in the respective domesticates. Skulls in the other columns represent brachycephalic varieties/breeds, according to the groupings as described in the text (“bulldog type,” “katantognathic,” and “allometric”). Numbers indicate discussed characteristics of the brachycephalic phenotype. It is evident that not all domestic species are represented by brachycephalic varieties and that the phenotype that is usually termed “brachycephalic” is variable in the different species. From left to right and top to bottom: Angora rabbit (Zoologisches Institut/Populationsgenetik [former Institut für Haustierkunde], Christian-Albrechts-Universität zu Kiel, Germany; I.f.H. 6489, mirrored); Polish rabbit (“Hermelinkaninchen,” I.f.H. 5348); domestic cat of unknown breed (I.f.H. 12689); Persian cat (I.f.H. 20428, mirrored); domestic dog of unknown breed (Paleontological Institute and Museum, University of Zurich; PIMUZ A/V 608); Boxer (PIMUZ A/V 2836, mirrored); Chihuahua (Albert Heim collection at the Naturhistorisches Museum Bern, Switzerland; NMBE 1052001); domestic pig of unknown breed (Zoological Museum, University of Zurich; ZMZH 17676); brachycephalic domestic pig of unknown breed (Nehring-Collection [Zoologische Sammlung der Königlichen Landwirtschaftlichen Hochschule zu Berlin] at the Museum für Naturkunde Berlin, Germany; ZMB_Mam_106884); domestic cattle of unknown breed (PIMUZ A/V 2, mirrored); Niata cattle (Natural History Museum of Denmark; NHMD-ZMK-MK-1109, mirrored; courtesy Kristian Murphy Gregersen); mixed breed goat (Center of Natural History, University of Hamburg; ZMH 10895, mirrored); and “Egyptian goat” (“Ägyptische Ziege”; Naturmuseum Wien, Austria; NMW 2074). “Normal” skulls are scaled to the same length across species and brachycephalic skulls are scaled to the non-brachycephalic ones of the same species; scale bars equal 1 cm. Specimens are dentally mature, except the brachycephalic pig. Cattles are shown with (graphically) cut horns. Erratum concerning figure 1e in Veitschegger et al. (2018): the schematic depiction of a brachycephalic cat skull (modified from Schlueter et al. 2009) shows a Persian cat, not a Siamese cat.

In the following, we give a general overview of the different domestic species and the varieties/breeds in which an extensive shortening of the snout has been reported and is considered to be occurring relatively consistently in many individuals or is even breed defining, i.e., where this condition is usually considered not just an occasional (random) variation in that variety/breed, e.g., as a pathology. These domestic species and the corresponding brachycephalic varieties and breeds are outlined in Table 2 and match in many cases with what has previously been described as “brachycephalic.” An overview of the domestic species, together with a categorization of types of brachycephaly (as outlined in the section “Types of brachycephaly”), is given in Fig. 1. Here, we focus on “ancient” domesticates that have been domesticated >500 years ago (Larson and Fuller 2014). Brachycephaly is—as far as we know—not reported from the more recent domesticates, such as e.g., mink, red fox, Syrian hamster, and chinchilla. Scientific names of domestic species in this review are according to Zeller and Gottert (2019), based on Bohlken (1961).

**Table 2:** List of ancient domesticated species in which breeds/varieties with extensive shortening of the snout, i.e., brachycephaly, are known and in which such a phenotype is not just occasionally occurring

Species	Brachycephalic varieties	References
Rabbit	In general, “dwarf rabbits” such as:- Polish- Netherland Dwarf- “Dwarf Rex” (Rexzwerg)- “Dwarf fox” (Fuchszwerg)	this study
Cat	An extensively shortened and dorsally rotated snout, associated with health issues, has mainly been described for two breeds of cats:- Exotic Shorthair- Persian	(Schlueter et al. 2009; Schmidt et al. 2017; Anagrius et al. 2021)
Dog	The following breeds have been described as brachycephalic according to their cranial proportions, dorsal rotation of the snout, and prevalence for diseases associated with brachycephaly and airorhynchy:- Affenpinscher- Border Terrier- Boston Terrier- Boxer- Brussels Griffon- Bulldog- Bullmastiff*- Cavalier King Charles Spaniel- Chihuahua- Dogue de Bordeaux- King Charles Spaniel/English Toy Spaniel- French Bulldog- Japanese Chin- Maltese- Miniature Pinscher- Pekingese- Pomeranian- Pug- Shih Tzu- Staffordshire Bull Terrier- Yorkshire Terrier	(Brehm et al. 1985; Koch et al. 2003, 2012; Schoenebeck et al. 2012; Packer et al. 2015a, 2015b; Marchant et al. 2017)*Unpublished data
Pig	The following varieties/breeds could be categorized as brachycephalic based on the description of their head configuration:- Neijiang: of China. The snout is short and snub-nosed.- Middle White: of England. Extremely short head with strongly dished and “squashed” profile.- Small White (Small Yorkshire): of England, now extinct. This breed's face has been described as very short and extremely dished (even “squashed”), with a broad and up-turned snout.	(Cheng 1985; Porter 1993; Sambraus 2001)
Cattle	Niata (Ñata): from South America, now extinct. Marked shortening and dorsal rotation of the snout relative to the braincase.	(Darwin 1878; Veitschegger et al. 2018)
Goat	Following goat varieties have been described to exhibit a pronounced convex nasal profile, i.e., roman nose. (Note that the presence of a roman nose is also described for other goat varieties, but reportedly not as marked). Additionally, anecdotal evidence suggests that an overshot lower jaw may not be a rare characteristic, although it is defined as an error in some breeding standards. There is probably a connection between the Damascus and the Zairaibi of Upper Egypt and possibly with the Indian dairy breeds.- Anglo-Nubian: English breed, developed mainly from the Jamnapari and the Zairaibi, crossed with European breeds. Today, individuals of this breed may still have the Zairaibi's undershot jaw, but the lower teeth should not be visible.- Beetal: of arid and semiarid Northwestern India. Reminiscent of the Nubian type with roman nose, but not as prominent as in Jamnapari breed.- Bhuj: of Brazil. Similar to the Beetal; mix from Indian breed(s) and Nubian.	(Acharaya 1982; Mason 1984; Porter 1996; Sambraus 2001; Khan and Okeyo 2016)
	- Jamnapari (Etawah): of arid and semiarid Northwestern India. Like the Beetal with a strongly convex profile giving it a “parrot mouth” such as seen in the Anglo-Nubian. One of the largest breeds in India.- Kamori: of Pakistan. With massive head and distinct roman nose.- Shami (Damaskus, Aleppo, Baladi, Damascene): of Syria and Lebanon.- Zairaibi (Egyptian Nubian, Theban): of Egypt. Strongly arched profile with lower lip that often projects beyond the upper, exposing its front teeth (undershot jaw).	

Note that due to the difficulties with defining varieties/breeds, intra-breed variation, and variable definitions of brachycephaly, this is not an exhaustive list.

In this discussion on brachycephalic varieties and breeds (especially also regarding Table 2), four main limitations regarding categorizations should be kept in mind: First, the definition of “breed” is ambiguous (Clutton-Brock 1999), especially if there are no breeding societies to define breeding standards and to ensure that breeds and their characteristics are maintained/bred pure (Acharaya 1982; Porter 1996). Therefore, we are referring to domestic subpopulations as “variety/breed” in the current paper. Second, individual variation within varieties/breeds is the norm, also in “well-defined” and purebred breeds (e.g., Epstein 1971; Nussbaumer 1982; Koch et al. 2012; Marchant et al. 2017). As a consequence, if a certain variety/breed is categorized brachycephalic, this might not apply to all individuals of that variety/breed. Third, there might be substantial variation of skull shape among subpopulations of varieties/breeds, as breeding standards and their interpretation may differ among countries and breeding clubs (Adametz 1926; Noden and Evans 1986). Fourth, breeding standards and customs, and subsequently head shapes of varieties/breeds of domestic animals, may be subject to change over historical time periods (e.g., Herre 1938; Nussbaumer 1982; Herre and Röhrs 1990; Drake and Klingenberg 2007; Geiger and Sánchez-Villagra 2018). Therefore, a breed or a variety that has traditionally not been considered brachycephalic may exhibit typical brachycephalic head features in the present times, or vice versa.

### Brachycephaly in the domestic carnivorans

Three members of Carnivora are regarded as ancient domesticates (Thomson 1951; Larson and Fuller 2014), two of which contain brachycephalic varieties/breeds: the dog (*Canis lupus* Linnaeus, 1758 f. familiaris) and the cat (*Felis silvestris* Schreber, 1778 f. catus) (Fig. 1; the third one being the ferret [*Mustela putorius* Linnaeus, 1758 f. furo], in which brachycephaly occurs only occasionally; Rempe 1962). The study of brachycephaly in cats and dogs has largely been focused on associations of the condition with a range of diseases that pose a considerable welfare issue in extreme varieties (Bessant, Sparkes and Rowe 2018) (see also later).

#### Domestic dogs

**Fig. 2:**
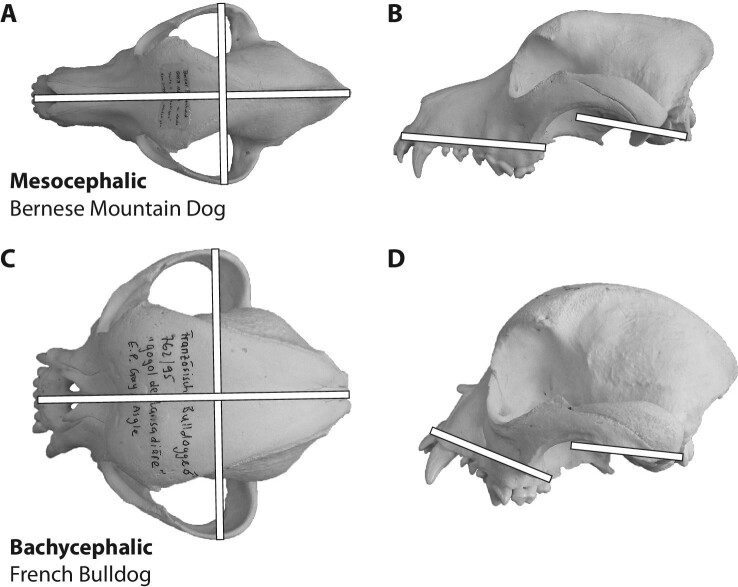
Schematic depiction of systems to discern brachycephalic from non-brachycephalic dogs. Different systems have been suggested to distinguish mesocephalic/mesaticephalic **(A and B)** from brachycephalic **(C and D)** domestic dogs. For example, indices of skull length and width (white bars in panels A and C) can be used to quantify the relatively short and broad skulls of brachycephalic varieties/breeds. Further, the angle between cranial base and palate (white bars in panels B and D) can be used to quantify the dorsal rotation of the snout; angles >180° are indicative of airorhynchy. Skulls are scaled to the same length and are housed in the collection of the Albert Heim Foundation at the Naturhistorisches Museum Bern, Switzerland: A and B, NMBE 1050197; C and D, NMBE 1051908.

In domestic dogs, brachycephaly has been defined as “short, wide headed” (e.g., Pekingese), as opposed to “dolichocephaly” which is defined as “narrow headed” (e.g., Collie), and “mesaticephaly”/“mesocephaly,” which is a head of “medium proportions” (e.g., German shepherd) (Ellenberger and Baum 1891; Lüps 1974; Evans 1993) (Table 1). Various systems have been suggested for a quantitative categorization of brachycephaly, including different indices of facial, neurocranial, and skull length and width dimensions in dry skulls or living individuals (e.g., Dahr 1941; Stockard 1941; Brehm, Loeffler and Komeyli 1985; Evans 1993; Koch et al. 2012) (Fig. 2).

The angle between cranial base and facial part of the cranium is widely used as another categorization system for brachycephaly in dogs. Generally, “airorhynchy” describes a state where the snout (measured at the palate) is rotated dorsally with respect to the cranial base. As a consequence, the angle between palate and cranial base is greater than 180° (Table 1; Fig. 2). On the other hand, “klinorhynchy” (also “clinorhynchy”) describes a ventrally rotated snout (Hofer 1952; Hofer 1960; Nussbaumer 1982; Baxter and Nussbaumer 2009). The quantification of airorhynchy can be conducted using crania or radiographs of living individuals (Regodón et al. 1993; Koch et al. 2003). Although the terms airorhynchy and klinorhynchy have originally been coined to describe skull conformations of wild mammals and birds, with the notion that these conformations are not equivalent to “pug-headedness” (Mopsköpfigkeit), i.e., brachycephaly, in domestic mammals (Hofer 1952), these terms are now ubiquitous when describing head shapes in domesticates. Moreover, “simognathy” has been described in some dogs, which is a condition that increases the appearance of a dorsal rotation of the snout via an additional dorsal rotation of the premaxillary relative to the palate (Selenka 1898; Rosenberg 1965; Nussbaumer 1982) (Table 1).

These different systems and ways to quantify brachycephaly (and airorhynchy) have complicated the meaningful categorization of head types in domestic dogs. Moreover, there usually is considerable variation among the individuals of a variety/breed concerning metrics associated with the categorizations (Nussbaumer 1982; Marchant et al. 2017). Further, given that body mass can differ as much as 40-fold among domestic dog breeds and that allometric scaling markedly influences skull morphology in domestic dogs (Klatt 1913), categorizations of breeds require taking into account body size. Notably, some dog breeds that are categorized as brachycephalic according to their relative skull dimensions (Brehm et al. 1985) do not necessarily exhibit airorhynchy (Thenius 1970; Nussbaumer 1982). For example, Pomeranian, Maltese, and Chihuahua have a reported mean prebasial angle of 168–172°, with no record above 176°, i.e., they are all non-airorhynchic (according to Nussbaumer 1982 and this study, Fig. 1; for data, see Table S1). As we will point out in more detail later, the short snout in these breeds seems related, at least to some extent, to small body size and allometric scaling (Klatt 1913; Lumer 1940; Lüps 1974; Rizk 2012; Cardini and Polly 2013).

In light of these issues and the continuous nature of brachycephaly, a categorical classification of brachycephaly on the basis of indices and thresholds is probably not warranted. Studies on overall skull shape across different dog breeds, although not providing guidelines for defining brachycephaly, point out sections in dog skull shape morphospace, where the brachycephalic skull shape conformation begins to appear in the continuum of face lengths in wild canids, modern breeds, and presumptive ancestral forms (Morey 1992; Morey 1994; Coppinger and Schneider 1995; Drake 2011; Marchant et al. 2017). However, also in such a sophisticated quantitative framework, categorizations of cranial shapes are problematic. In summaries of multivariate spaces, it might be tricky to pinpoint locations in shape space where transitions between categories occur, i.e., one might overinterpret overlap, or the lack of it. Domestic dog breeds that are typically classified as brachycephalic based on their skull proportions (also on a continuous scale), airorhynchy, and prevalence for certain brachycephaly-related diseases are listed in Table 2. (Note that this list may not be exhaustive, given the described issues concerning definitions).

#### Domestic cats

In domestic cats, increasing degrees of brachycephaly (from mild to severe) have been characterized qualitatively by an increasingly more pronounced horizontal orientation of the upper canine teeth, dorsal rotation of the jaws (airorhynchy, Table 1), pronounced angle between the nasal and frontal bones (“stop”), relatively small facial bones (maxillary and nasal), and a rounded (dome-shaped) braincase (Künzel, Breit and Oppel 2003; Schlueter et al. 2009; Farnworth et al. 2018).

More quantitative ways to grade brachycephaly in cats are based on rhinarium size, degree of stenotic nares, type of nares, and the alignment of the eyes and the rhinarium in lateral or frontal view (Schmidt et al. 2017; Anagrius et al. 2021). The latter categorization has been used to discern between “normal,” i.e., wild-type like cats, “doll-face” Persian cats, with relatively low grade brachycephaly, and “peke-face” types (Schmidt et al. 2017). The severe-grade peke-face phenotype (name derived from the similarly looking, flat-faced Pekingese dog) is characterized by a sphere-like (short, broad, high, and round) braincase, marked reduction of the size of the nasal bones, flat orbits, a prognathic mandible with dental malocclusion, dorsal rotation of the canines and incisors, and absence of the frontal sinuses and retrograde growing conchae (Schmidt et al. 2017).

Exotic Shorthair and Persians are the most extreme examples of facial shortening in cats and usually categorized as brachycephalic breeds (Table 2). Selection for a roundish and rather flat face also exists in other breeds or subpopulations/strains of these breeds, e.g., a strain of the Burmese cat in the United States, in which the brachycephalic phenotype is linked with lethal malformations (Noden and Evans 1986; Lyons et al. 2016). Anecdotal evidence based on examination of breeding standards points into a similar direction in lines of American (shorthair and wirehair), Bombay, British (shorthair and longhair), Himalayan, Scottish fold, and Selkirk Rex (American Cat Fanciers Association; Governing Council of the Cat Fancy; The Cat Fanciers' Association; Gunn-Moore, Bessant and Malik 2008).

### Brachycephaly in the domestic artiodactyls

“Artiodactyla” (even-toed ungulates plus whales) include many domesticated species (Larson and Fuller 2014), including Bactrian camel and dromedary (*Camelus ferus* Przewalski, 1878 f. bactrianus and *C. ferus* Przewalski, 1878 f. dromedarius), llama (*Lama guanicoe* Statius Müller, 1776 f. glama), alpaca (*Vicugna vicugna* Molina, 1782 f. pacos), pig (*Sus scrofa* Linnaeus, 1758 f. domestica), reindeer (*Rangifer tarandus* Linnaeus, 1758 f. domestica), goat (*Capra aegagrus* Erxleben, 1777 f. hircus), sheep (*Ovis orientalis* Gmelin, 1774 f. aries), water buffalo (*Bubalus arnee* Kerr, 1792 f. bubalis), taurine and indicine cattle (*Bos primigenius* Bojanus, 1827 f. taurus and *B.primigenius* Bojanus, 1827 f. indicus), yak (*Bos mutus* Bojanus, 1827 f. grunniens), Bali cattle (*Bos javanicus* d'Alton, 1823 f. domestica), and gayal (*Bos gaurus* Smith, 1827 f. frontalis) (Fig. 1). To the best of our knowledge, brachycephalic varieties/breeds are only described in the domestic pig, taurine cattle, and goat (Table 2). In some of the other domestic species, a shortening of the maxilla leading to mandibular prognathism might occur as an occasionally occurring pathology.

**Fig. 3:**
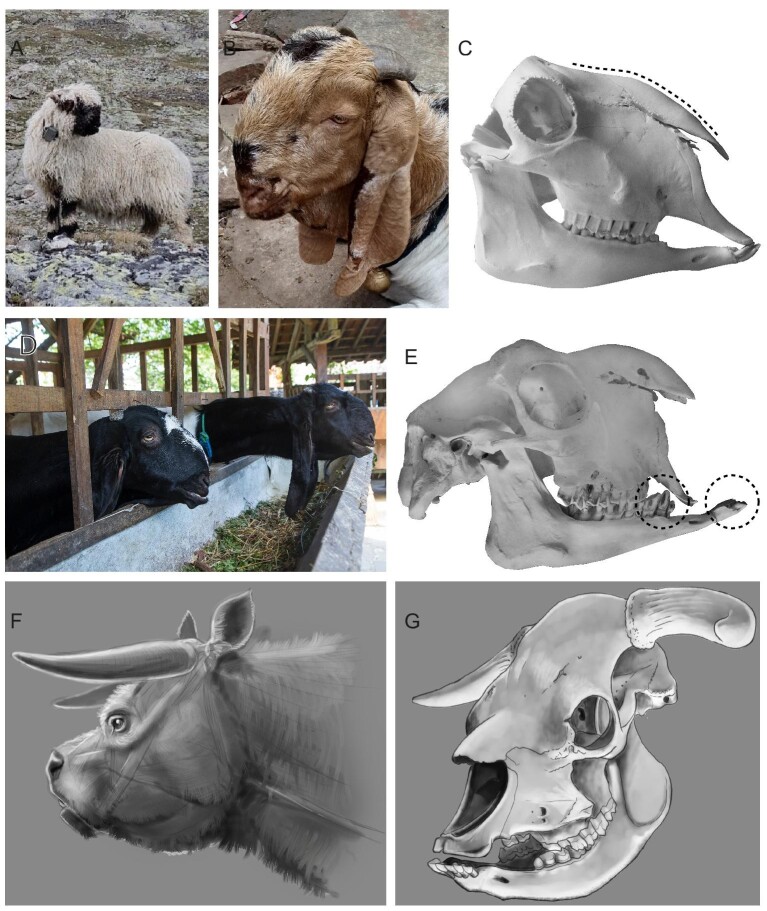
Facial shape variation and brachycephaly in domestic ruminants. Certain varieties and breeds of sheep (**A**; Valais Blacknose sheep) and goats (**B**; breed unknown, Bangalore, India) exhibit a convex profile of the nose, which is termed “roman nose” (shown as a dashed line in panel C). These variations are mostly relatively mild and do not result in discordance between maxilla and mandible length, as shown on the example of the skull of a Valais Blacknose sheep (**C**; Musée de la Nature du Valais, Switzerland; HN 2010511). However, in certain goat varieties and breeds, such as Jamnapari/Etawah goats **(D)**, extreme “roman nose” may be associated with an overshot lower jaw and dental malocclusion. The overshot lower jaw and dental malocclusion (dashed circles in panel E) are shown on the example of the skull of an “Egyptian goat” (**E**; “Ägyptische Ziege”; Naturmuseum Wien, Austria; NMW 2074). These varieties/breeds could be classified as “katantognathic” brachycephalic, where, in addition to the extremely convex nasal bones, parts of the snout (premaxilla) are foreshortened and downward tilted (Fig. 1 and Table 1). In other domestic ruminants, such as cattle, no cases of “katantognathic” brachycephaly are known. Instead, the extinct Niata cattle from South America (**F**, reconstruction) is characterized by shortened and upward tilted facial bones **(G)**, which is indicative of “bulldog-type” brachycephaly (Fig. 1), and may also lead to dental malocclusion (G). Pictures are not to scale. Credits: A, Benjamin Jost; B, C, E: Madeleine Geiger; D, Shutterstock: Ibenk_88; F, G: Artwork by Jorge González.

Head shape in artiodactyl domesticates varies widely, from a concave profile of the nose (“dished face,” e.g., Somali goat, Algarvia goat; Porter 1996) to a convex profile of the nose (“roman nose,” e.g., Vallais Blacknose sheep; Acharaya 1982; Hendricks 1995; Porter 1996), depending on the variety/breed in question. Although these shape variations are mostly relatively mild and do not results in discordance between maxilla and mandible length, extreme “dished faces” have been described for particular pig and cattle breeds (e.g., Middle White and Niata cattle, respectively) and an extreme roman nose is characteristic for particular goat breeds (e.g., Egyptian goat) (Table 2; Figs. 1 and 3). These varieties, sometimes exhibiting an overshot lower jaw, have been termed brachycephalic (see later and Table 2).

Brachycephaly in cattle, pigs, and goats does not seem to be correlated with body size. Brachycephalic Niata cattle have been reported to be of average size compared with other taurine cattle (Veitschegger et al. 2018). Brachycephalic goats are often described as large animals (Acharaya 1982) and also brachycephalic pigs are not particularly small. The most famous brachycephalic pig breed is the Middle White, whose name describes its average body size compared with that of related breeds (Porter 1993).

Apart from the abovementioned varieties, brachycephaly has been described to occur in association with disproportional dwarfism (chondrodysplasia), reported in some sheep breeds (e.g., Cabugi, Texel, Cheviot, Suffolk, Hampshire, and Merino) and cattle breeds (e.g., Dexter, Horned Hereford Dwarf, Aberdeen Angus) that are homozygous for certain genetic variants that are considered pathological and therefore undesirable (e.g., Julian et al. 1957; Grüneberg 1963; Thompson et al. 2005; Cavanagh et al. 2007; Thompson, Piripi and Dittmer 2008; Dantas et al. 2014; Boegheim et al. 2017).

#### Domestic pigs

In domestic pigs, head shape varies considerably among varieties/breeds (Owen et al. 2014). The face and snout may be long and straight, short and convex (“dished” and with “snub nose”), and everything in between (e.g., Porter 1993; Sambraus 2001). Brachycephalic pigs (Table 2) are characterized by a short and broad head with relatively short and dorsally rotated facial bones (airorhynchy, Fig. 1; Table 1). The nasal bones are concave. It is assumed that the short nose and concave profile of these pig varieties is an original characteristic of some Chinese breeds (Porter 1993).

Besides a few breeds with extensive brachycephaly and airorhynchy (Fig. 1; Table 2), some domestic pig breeds may exhibit a tendency toward airorhynchy or simognathy without extensively shortened snouts or only subpopulations/strains within these breeds exhibiting such phenotypes. Examples include Kunekune, Yorkshire, Berkshire, Kolbroek, Göttinger Minischwein, and Vietnamese pot-bellied pigs. (Note that this has not been studied quantitatively so far and that this is a qualitative statement based on visual examinations of skulls and photographs.)

#### Domestic cattle

Brachycephalic cattle have been described to exhibit short premaxillary and maxillary bones with a relatively short diastema, short nasal conchae, short and convex nasal bones, circular alignment of the cheek teeth, a curved and overshot lower jaw, and airorhynchy (for a detailed description, see Veitschegger et al. 2018) (Figs. 1 and 3).

There are various cattle varieties/breeds in which brachycephalic specimens are known to have occurred. One of the most pronounced brachycephalic varieties/breeds is the South American and now extinct Niata cattle (Table 2; Fig. 3). Other cattle varieties, except from the lethal ones described earlier, may exhibit relatively short snouts (e.g., Tuxer, Zillertaler). Further, in the Jersey cattle and the Swiss Braunvieh and Simmenthaler cattle, specimens with a brachycephalic and airorhynchic head shape have been described (Adametz 1926; Duerst 1931; Becker and Arnold 1949; Veitschegger et al. 2018). However, the Niata's skull shape is by trend more extreme compared with these other cattle breeds, with more pronounced brachycephalic features (Darwin 1878; Becker and Arnold 1949; Veitschegger et al. 2018). Moreover, contrary to the Jersey and Braunvieh specimens, the brachycephalic phenotype appears to have been occurring relatively consistently in most individuals of the Niata and was not just an occasional variation in that variety/breed (Veitschegger et al. 2018). However, the breed status of the Niata is questionable to this day and the occurrence probably the result of a small founder population (Veitschegger et al. 2018).

#### Domestic goats and sheep

In domestic sheep, convex nasal profiles are exhibited by many varieties/breeds worldwide to various degrees, with only slight arching (e.g., Meat Merino) to a more prominent convexity (e.g., Valais Blacknose sheep, Fig. 3) (e.g., Acharaya 1982; Sambraus 2001). However, such roman noses are usually not considered brachycephalic per se (see earlier).

The same is true for domestic goats. However, in contrast to sheep, some goat varieties/breeds exhibit quite strongly bulged nasal profiles, with the lower jaw projecting beyond the upper and exposing the lower incisors and canines (Porter 1996). Such extreme goats (Table 2; Figs. 1 and 3) have been described as exhibiting a triangular head shape, reminiscent to the one of a pug (“Mopskopfbildung”; Herre and Röhrs 1990).

The skull of brachycephalic goats is characterized by short and convex nasal bones, and short premaxillary and maxillary bones. While the premaxilla is ventrally rotated relative to the palate (“katantognathy”; Table 1), there is no apparent change in the angle between the cranial base and the palate (i.e., no airorhynchy; Table 1). Note that this has not been studied quantitatively so far and that this is a qualitative statement based on visual examinations of a few rare skulls in museum collections (Figs. 1 and 3).

Brachygnathia superior (Table 1) has been described to be a birth defect occurring more frequently in goat breeds selected for a roman nose, especially if the convexity is pronounced (Al-Ani et al. 1998). These brachycephalic goat varieties/breeds with convex nasal profiles and long ears (Table 2) are predominant in North-East Africa (Egypt and Sudan), West Asia (Syria and Lebanon), and parts of the Indian subcontinent (North India and Pakistan) (Porter 1996).

### Brachycephaly in the domestic rodents and lagomorphs

Three Glires species are considered ancient domesticates (Berry 1984; Larson and Fuller 2014): the house mouse (*Mus musculus* Linnaeus, 1758 f. domestica), the guinea pig (*Cavia aperea* Erxleben, 1777 f. porcellus), and the rabbit (*Oryctolagus cuniculus* Linnaeus, 1758 f. domestica) (Fig. 1). Only in certain strains, varieties, and breeds of rabbits is an overshot lower jaw a relatively frequently occurring malformation (see the next section), leading to dental malocclusion, while this condition seems to be occurring occasionally in guinea pigs (Studer 1975; Müller et al. 2015). Brachycephaly is also known from genetically modified strains of mice, which are, however, beyond the scope of this review (e.g., Hajihosseini et al. 2001).

The smallest among the domestic rabbit breeds exhibit short snouts relative to the braincase and could probably be classified as brachycephalic on the basis of their relatively short snout compared with larger forms, while the braincase scales proportionally with size (“allometric” brachycephaly; Klatt 1913; Fiorello and German 1997) (see later and Fig. 1). (Note that there has been no study explicitly assigning the term “brachycephalic” to these dwarf breeds.) An overall relatively short snout is different from brachygnathia superior, i.e., a disproportionate shortening of the upper jaw relative to the lower one (Table 1), which is generally considered a pathology in domestic rabbits (e.g., Van Caelenberg et al. 2008) and not a recognized characteristic of any rabbit variety or breed (Hückinghaus 1964). However, mainly (but not exclusively) “dwarf rabbits” with less than 1.5 kg body weight and about 3.5 cm ear length have been described as being prone to brachygnathia superior, which is regarded as synonymous to brachycephaly (e.g., Schnecke 1941; Böhmer 2003; Verstraete and Osofsky 2005; Reiter 2008) (Table 1). Dwarf rabbit breeds include, e.g., Polish and Netherland dwarf rabbits (Table 2). However, disproportionate shortening of the upper jaw has also been reported for some strains of different (not only dwarf) rabbit breeds in the lab (Fox and Crary 1971; Huang, Mi and Vogt 1981). Although it has been shown that the angle between face and the braincase is variable among pet rabbits, rabbits in general are characterized by klinorhynchy (Böhmer and Böhmer 2020), and to our knowledge, airorhynchy or katantognathy (Table 1) have not been reported in any rabbit variety or breed.

### Brachycephaly in other domestic animals

Varieties with a particular short face are also known from nonmammalian domesticates. Examples for pigeons include English Short-Faced, African Owl, Long-Face Clear Leg, Blondinette, Helmet, and Modena pigeons (Young et al. 2017). One example of a chicken with particularly short beak is the Kilimookku Aseel (long-tailed parrot beak Aseel). Extremely short beaks in these birds, however, do not appear to cooccur with mandibular prognathism, as is typical for many mammals described as brachycephalic. The underlying developmental events and skeletal changes that lead to shortening of the face in birds are at least partially distinct from those occurring in other amniotes. This is because facial length in birds is dependent almost entirely on evolutionary variation in the size of the premaxilla, whereas the maxilla remains quite small; and this is in contrast to the skulls of other amniotes where facial length is almost always determined by evolutionary variation in the size of the maxilla (Young et al. 2014). On the other hand, evidence for similar allometric scaling relationships of the facial skull as in mammals (cranial evolutionary allometry hypothesis or “CREA”; Cardini 2019) have also been found in birds (Bright et al. 2016; Linde-Medina 2016; Tokita *et al.* 2017).

In teleost fishes, a bulged appearance of the skull and the head has been described in some varieties of goldfish, e.g., Ranchu and Lionhead (Dobkowitz 1962; Hans 2002), where disproportionate growth of the upper or the lower jaw appears to be absent. In carps, occasional occurrence of “bulldog-headed” individuals has been recorded, with the lower jaw being of normal length but the face ending abruptly in front of the eyes (Bateson 1894). As the bony elements of the amniote skull are apomorphic, i.e., highly derived from the conformation as present in teleost fishes, the underlying developmental process resulting in superficially similar brachycephalic phenotypes is probably substantially different and an example of convergence.

## Types of brachycephaly

**Fig. 4:**
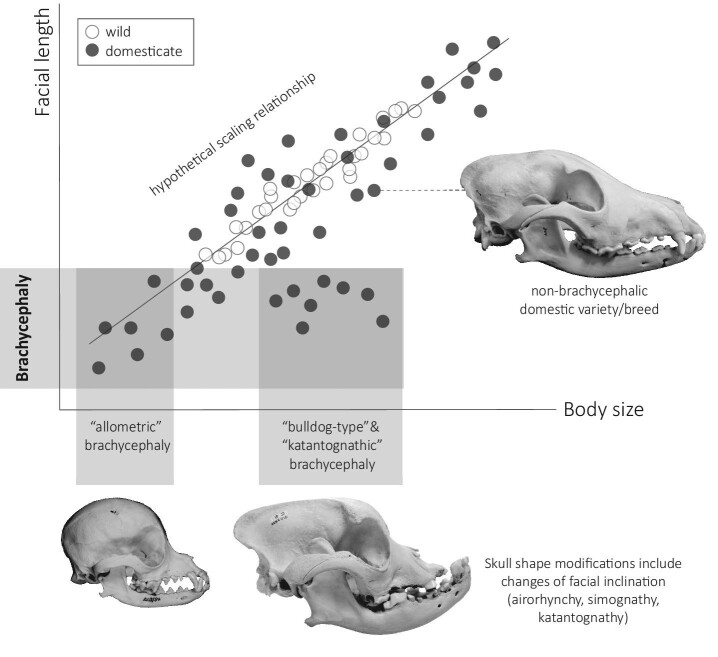
Hypothetical scaling relationship between body size and facial length in any wild animal (white dots) and its domestic counterpart (black dots). The latter exhibit larger intragroup variation of body size and facial length, visualized via more scattering of dots along the common scaling axis (straight line). This comparison exemplifies the difference between “short snoutedness,” i.e., brachycephaly (black dots incorporated into the horizontal box), due to small size (“allometric” brachycephaly) and due to shortening of facial bones not directly resulting from small body size (“bulldog-type” brachycephaly or “katantognathic” brachycephaly). The latter is usually associated with skull modifications, including changes of facial inclination, whereas the former is not per se. Brachycephalic skull proportions may not occur in the respective wild forms. Photographs of skulls depict domestic dogs as an example (for details on specimens, see Fig. 1). The skulls are to scale.

What the earlier descriptions make apparent is that across time, species of interest, and research fields (e.g., veterinary medicine, evolutionary morphology, domestication research), the term “brachycephalic” remains highly variable in its definition and use. Phenotypes that are referred to as “brachycephalic” include a range of different morphological characteristics of the skull among different domestic species, either in isolation or combination, and in various degrees of expression (Fig. 1). In other words, the term “brachycephaly” may be regarded a homonymy, where the same term is used to describe potentially inherently different states. Therefore, there is a need to discern different types of brachycephaly. These types are not necessarily mutually exclusive and they do not imply similarity or difference of underlying genetic and developmental mechanisms. They should merely facilitate the morphological categorization of skull shapes. A summary of the concept of these three morphotypes of brachycephaly and how they relate to body size is shown in Figs. 1 and 4.

### “Bulldog-type” brachycephaly

In some brachycephalic varieties/breeds of dog, cat, cattle, and pig, a disproportional shortening of the facial bones appears to cooccur with an upward tilting of the snout relative to the rest of the skull, a condition typically found in bulldogs (Fig. 1). Such inclinations may include airorhynchy (dorsal rotation of the palate relative to the cranial base) and simognathy (dorsal rotation of the premaxilla relative to the palate and the maxilla) (Fig. 1; Table 1). There is at least in part a genetically founded correlation (see later) of this kind of brachycephaly with overall body size in domestic dogs, with a tendency of “bulldog-type” domestic dog breeds to be on the small side of the domestic dog body size spectrum (Marchant et al. 2017; Fig. 1G, regression of body size [neurocranium centroid] as the independent variable and viscerocranium shape as the dependent variable, *r*^2 ^= 0.889), although airorhynchy is also known from medium-sized and giant breeds, such as the Boxer and Dogue de Bordeaux (Nussbaumer 1982; Marchant et al. 2017). Besides these genetic factors, there is also likely to be involvement of spontaneous mutations, the unpredictable effects of hybridization, and breeding practice, which might tend to promote a “bulldog-type” brachycephalic phenotype in toy breeds, or counter-select this phenotype in larger breeds of dogs. Apart from dogs, “bulldog-type” brachycephaly does not appear to be correlated to body size. The airorhynchic Niata cattle have been reported to be of average size compared with other taurine cattle (Veitschegger et al. 2018), and also the airorhynch Middle White pig is not a particularly small pig breed (Porter 1993). In sum, underlying mechanisms of “bulldog type” brachycephaly seem to be multigenic and far from simple, even within just one domestic species (i.e., dogs), and thus potentially even more so in the other, so far less well-investigated domesticates.

### “Katantognathic” brachycephaly

Like “bulldog-type” brachycephaly, “katanognathic” brachycephaly is associated with an unusual inclination of the facial bones relative to the rest of the skull (Fig. 1). However, in contrast to the “bulldog-type” airorhynchy and simognathy, this type of brachycephaly is characterized by katantognathy, which is the ventral rotation/downward tilting of the premaxilla relative to the palate and the maxilla (Table 1).

Among domesticated varieties, katantognathy is a feature of some goats (Figs. 1 and 3; Table 2) as well as some klinorhynchic domestic dogs, e.g., Bullterriers (Nussbaumer 1982). However, a concomitant shortening of the facial bones is only present in the goats, while in the bullterrier, the facial bones are of the same relative size as in the wolf (Nussbaumer 1982). Here, it has been argued that a shortening of the facial bones associated with klinorhynchy would likely be deemed unaesthetic in dogs (Nussbaumer 1982).

Not much is known about developmental pathways and genetic underpinnings of “katantognathic” brachycephaly, other than that it does not appear to be associated with small body size: brachycephalic goats, which show a ventral rotation of the premaxilla, have been described as “large animals” (Acharaya 1982).

### “Allometric” brachycephaly

Some small, or toy, varieties/breeds of dogs (e.g., Chihuahua, Pomeranian) and dwarf rabbits (e.g., Netherland dwarf) (Table 2) are characterized by short snouts in relation to the entire skull and/or the braincase, compared with larger varieties (e.g., Klatt 1913; Fiorello and German 1997) (Fig. 1). Two different patterns may contribute to this phenomenon, related to allometric relations of brain and facial length to body size (Fig. 4).

First, brain size and hence brain case volume scale negatively allometrically with body size in vertebrates (“Rule of Haller”; e.g., Klatt 1913; Gould 1975; Bauchot 1978; Bronson 1979; Radinsky 1985; Emerson and Bramble 1993; Lüps 2008). In other words, small-bodied mammals have relatively larger brains and neurocranial portions, which subsequently make up a larger portion of the entire cranial length (Fig. 4). The underlying reason for this allometry is probably physiological in nature (Epstein 1971 and references therein): to maintain all the body functions, the quantity of nervous substance cannot be reduced beyond a certain limit; additionally, the relatively larger body surface of small animals results into relatively more sensory cells on its surface, which again require the respective centers in the brain to process the signals.

Second, facial length scales positively allometrically with total cranial length or body size, both within domestic species, e.g., dogs (Lumer 1940 and references therein) and among closely related species of various mammalian clades, sharing a similar cranial bauplan (cranial evolutionary allometry hypothesis or “CREA”; Radinsky 1985; Emerson and Bramble 1993; Cardini and Polly 2013; Cardini et al. 2015; Tamagnini, Meloro and Cardini 2017; Cardini 2019; Le Verger et al. 2020) and birds (Bright et al. 2016; Linde-Medina 2016; Tokita *et al.* 2017). In other words, large species tend to have relatively longer faces than smaller ones (or the other way around: small species tend to have relatively shorter faces than larger ones; Fig. 4). The underlying reason for this may be dietary and biomechanical, as larger mammals need proportionally larger feeding apparatus to maintain function and efficiency (Gould 1975; Emerson and Bramble 1993; Slater and Van Valkenburgh 2009; Cardini and Polly 2013). On the other hand, scaling relationships deviating from CREA are known from a limited number of lineages, e.g., in African antelopes and equids (Cardini 2019). In these groups, palatal portions of the cranium scale isometrically or even positively allometrically with body size, leading to a relatively long ventral portion of the snout in small species (Cardini 2019). These scaling relationships in some grazers might reflect the need for relatively large hypsodont teeth and thus palate to process a greater quantity of food compared with dicot feeders (“long face hypothesis”; Spencer 1995; Cardini 2019).

Testing allometric scaling of cranial shape among varieties/breeds of domestic species would ideally entail the examination of closely related varieties/breeds (or even ancestral ones, if known), as allometric scaling patterns among clades in nature (i.e., Haller's rule and CREA) concern closely related species (Cardini 2019). However, due to extensive interbreeding of varieties throughout parts of the history of many domestic forms (e.g., dogs [Parker et al. 2017] and chicken [Núñez-León et al. 2019]), this will be notoriously difficult to achieve.

It has been shown that in African tree squirrels, smaller species have less straight snouts compared to larger species (Cardini and Polly 2013). However, in the abovementioned dwarf dogs and rabbits, there is no apparent and uniform angular change of any part of the face relative to other parts of the cranium compared with larger varieties, such as seen in “bulldog-type” and “katantognathic” brachycephaly (Rizk 2012). However, mandibular prognathism is a relatively frequent malformation in dwarf rabbits (see earlier) and “bulldog-type” brachycephaly (see earlier) may shape the cranium at the same time as “allometric” brachycephaly in some breeds of domestic dog, e.g., in the small and airorhynchic Pekingese and Shih-Tzu. Thus, “allometric” and “bulldog-type” brachycephaly may be regarded as different patterns (Epstein 1971; Rizk 2012), although there might be some shared genetic and developmental bases (see later).

## Pathological and morphological correlates of brachycephaly

Profound alterations of cranial morphology as seen in brachycephalic varieties are associated with a number of other morphological characteristics and even pathological conditions, which are discussed in the following paragraphs. These conditions have been particularly well studied in domestic dogs and cats and concern mostly, but not exclusively, the dentition and the upper airways. The high prevalence of pathological conditions in strains exhibiting extreme brachycephaly raises urgent questions concerning animal welfare and should be subject to open discussion considering adjustments of breeding standards and interpretations thereof.

**Fig. 5:**
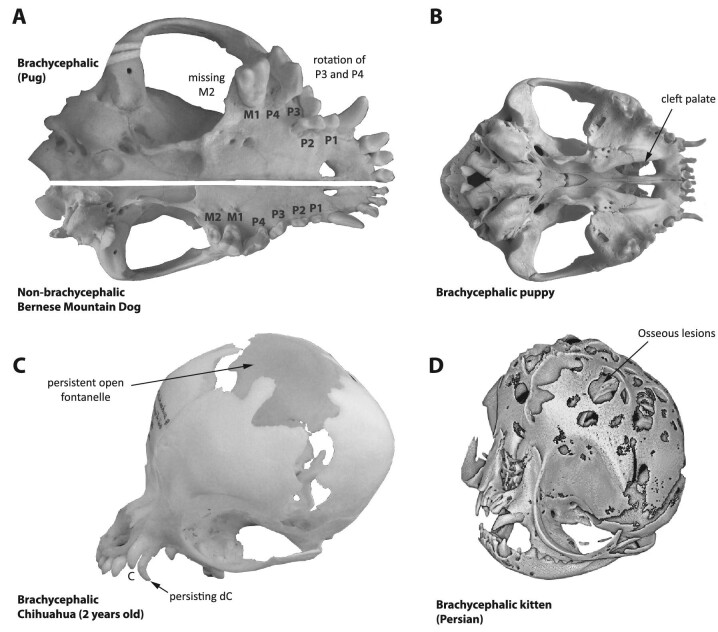
Examples of craniodental anomalies that may cooccur with “bulldog-type” and “allometric” brachycephaly. **(A)** An example of a brachycephalic pug (left ventral aspect of cranium; Naturhistorisches Museum Bern, collection of the Albert Heim Foundation, Switzerland; NMBE 1062021) showing crowding of the postcanine teeth and a rotation of the third and fourth upper premolars (P3 and P4) relative to the longitudinal axis of the cranium. Additionally, the second upper molar (M2) is missing (note that there is little space caudal to M1 to house such a tooth). As a comparison, the example of a non-brachycephalic Bernese Mountain Dog (right ventral aspect of skull; NMBE 1050197) below shows the wild-type dental formula and much less to absent dental crowding and rotation. **(B)** An example of a cleft palate (bony portion) in the cranium of a puppy of a bulldog (photo by R.A.S. of specimen from his personal collection). **(C)** An example of a 2-year-old Chihuahua (NMBE 1051992) exhibiting persistent open fontanelles and a deciduous canine tooth (dC), next to the permanent canine (C). Usually in dogs, the fontanelle fuses a few days or weeks after birth (De Lahunta and Glass 2009) and the deciduous canines are usually replaced by about a half a year of age (Habermehl 1975). (**D**) Osseous defects in the parietal and frontal bones of a 5-day-old Persian kitten (Schmidt et al. 2017). Skulls are not to scale. Please note that this list of characteristics is not exhaustive. For more craniodental anomalies associated with brachycephaly, also including soft tissue, see text.

The most important health issue of “bulldog-type” brachycephalic domestic dogs and cats with high impact on the welfare of these animals is brachycephalic airway obstruction syndrome (BOAS). The reduction of the facial bones leads to a mismatch between bone and nasopharyngeal soft tissues causing increased upper airway resistance, respiratory distress and exercise intolerance (Knecht 1979; Packer et al. 2015a, 2015b). Although the nasal turbinals/conchae of brachycephalic dogs are smaller, simpler, and more loosely arranged than in non-brachycephalic ones, they are extremely densely packed and additionally, there is aberrant turbinal/conchal growth into the nasal passage and/or the choanae (Oechtering, Oechtering and Nöller 2007; Oechtering et al. 2016; Wagner and Ruf 2020). The mucus membranes of the nose are of vital importance for thermoregulation (Ginn et al. 2008); their reduction explains brachycephalic dogs’ and cats’ decreased capacity for thermoregulation and propensity for heat intolerance (Davis, Cummings and Payton 2017). Other findings regarding the oronasal system suggest that brachycephalic (including dwarf) domestic dogs exhibit greatly reduced or even absent frontal sinuses (Weidenreich 1941; Evans 1993). Further, cribriform plate shape has been found to be more rostrocaudally compressed and flattened in domestic dogs that tend toward brachycephaly, compared with dogs with a relatively longer snout (Jacquemetton et al. 2020). Relatedly, brachycephaly has been found to be associated with tympanic bulla malformations (Mielke, Lam and Ter Haar 2017) and a higher prevalence of orofacial clefts, especially cleft palate (Foley, Lasley and Osweiler 1979; Mulvihill, Mulvihill and Priester 1980; Moura, Cirio and Pimpão 2012; Moura, Pimpão and Almasri 2017; Roman et al. 2019) (Fig. 5). However, the prevalence of orofacial clefting in brachycephalic domestic dogs may be associated with shared ancestry as many brachycephalic breeds belong to terrier and mastiff groups, whose mesaticephalic members also exhibit a prevalence for orofacial clefting (Roman et al. 2019).

Reduction of the maxillary bone in “bulldog-type” domestic dogs leads to redundant skin with excessive folding on the nose ridge and dermatitis. Moreover, the maxillary bone offers less space for dental alveoli, which is why reduction of teeth (oligodontia, either congenial or acquired due to high prevalence of dental diseases as the result of malocclusion), crowding, and rotation related to alveolar processes are common, (McKeown 1975; Harvey 1985; Kupczyńska et al. 2009; Schlueter et al. 2009; Lobprise and Dodd 2019) (Fig. 5). As a result, dental occlusion is often disrupted and the carnassial complex, which in carnivorans consists of the upper fourth premolar and the lower first molar and which is used as scissor-like shearing complex, is misaligned (Selba et al. 2019). In dwarf breeds, dental reduction, crowding, and rotation are either due to a minimal tooth size that cannot be undercut and/or due to negative allometric scaling of tooth size; small varieties have relatively larger teeth than large ones, resulting in too little space for the full set of permanent teeth (Weidenreich 1941; McKeown 1975; Curth 2018). As far as the authors are aware, in domesticates other than dogs, similar tooth crowding due to small size and/or disproportionate shortening of the maxilla have not been reported. In domestic cats, this may be due to the extensive reduction of tooth loci in the course of felid evolution and thus less acute space problems (Ungar 2010). In domestic ungulates, crowding is probably prevented by the spare space provided by the diastema as well as the pronounced mesiodistal interlocking postcanine teeth. However, in domestic rabbits, malocclusion as the result of brachygnathia may lead to overgrowth of the ever-growing incisors and cheek teeth, which is often fatal (Wiggs and Lobprise 1997).

Brachycephalic dogs and cats have less sensory innervation in their cornea (Blocker and Van Der Woerdt 2001) and extreme shallow orbits. Both morphological features predispose to ocular proptosis and both to chronic corneal epithelial defects. The nasolacrimal duct system runs in a right-angled or even acute-angled inclination (Breit, Künzel and Oppel 2003; Schlueter et al. 2009) that is associated with inadequate drainage of the lacrimal fluid. The same holds for the eustachian tube. Kinking of this drainage leads to accumulation of fluid in the auditory bulla and secretory otitis media (Hayes, Friend and Jeffery 2010).

One consistent feature of brachycephalic dogs and cats is the shortening of the cranial base and a reduced cranial capacity that can cause overcrowding (i.e., a larger total brain volume relative to body weight) and brain herniation (Carrera et al. 2009; Schmidt et al. 2013, 2014; Selba et al. 2020; Sokołowski et al. 2020). It has been suggested that this volumetric restriction in brachycephalic dogs also leads to a more ventrally rotated longitudinal brain axis, i.e., progressive ventral pitching of the brain, as well as a more ventrally shifted olfactory bulb position (Roberts, McGreevy and Valenzuela 2010; Hussein, Sullivan and Penderis 2012). The volume overload has a severe impact on cerebrospinal fluid (CSF) flow. Laxity of the craniocervical junction, subluxation and “invagination” of the atlas into the foramen magnum contribute to the constriction at the level of the spinal canal, which further compromise the CSF flow (Cerda-Gonzalez et al. 2009). The reduced longitudinal expansion of the cranial base is in part compensated by a widening of the cranial base and a reduced volume of the jugular foramina and volume overload of the venous compartment. This in turn reduces CSF absorption from the subarachnoid space into intracerebral veins via pacchionian granulations. All these morphological alterations lead to a holding back of CSF in the cerebral ventricles resulting in accumulation of CSF and communicating hydrocephalus. A second consequence is turbulent CSF-flow patterns and increased CSF flow velocity at the craniovertebral junction that forces CSF into the central canal of the spinal cord leading to syringomyelia (SM) (Hu et al. 2012). This spinal cord disease leads to neuropathic pain, and if expansion of SM is not treated, to motor dysfunction and paralysis (Rusbridge 2005). However, SM is mainly found in a few breeds tending toward brachycephaly, such as Cavalier King Charles Spaniels, while it is not prevalent in many other brachycephalic breeds, which indicates that this condition might rather be associated with breed specific factors.

Dwarf/toy domestic dog breeds further exhibit specialties concerning their cranial bones and teeth. First, there is a relatively high incident of deciduous teeth—especially the upper canines—being retained into adulthood due to reasons unknown (Harvey 1985; Butković et al. 2001) (Fig. 5). Second, dwarf breeds—notably Chihuahuas—often exhibit persistent open fontanelles, i.e., bones of the cranial vault that do not fuse, even in adulthood (Kiviranta et al. 2021a, b). These persistent open fontanelles are probably linked to these dogs’ extreme dwarfism and corresponding relative large brain size while the bones of the cranial vault are scanty (Weidenreich 1941; De Lahunta and Glass 2009) (Fig. 5). On the other hand, persistent open fontanelles also appear to be related to Chiari-like malformation and syringomyelia and thus abnormal skull shape and growth (Kiviranta et al. 2021a, b). Osseous lesions of calvarial bones are known from brachycephalic cats (Schmidt et al. 2017) (Fig. 5).

**Fig. 6:**
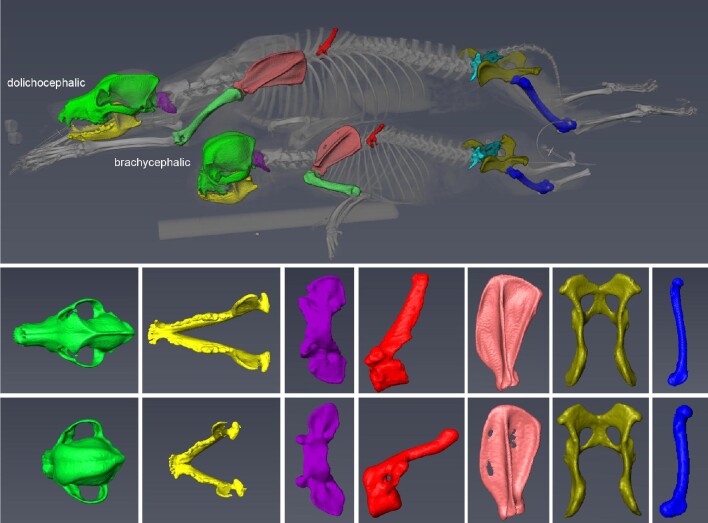
“Bulldog-type” brachycephaly and its relation to the postcranial skeleton. Although brachycephaly most conspicuously concerns the facial part of the cranium (green) and the mandible (yellow), it may also be correlated with shape variation of the vertebrae (purple, red; note that the brachycephalic dog exhibits a vertebral malformation), scapula (pink), pelvis (olive), and the long bones of the limbs (femur, blue). Most of these bones are stouter in the brachycephalic than in the non-brachycephalic varieties/breeds. Genetic and developmental processes affecting head shape in “bulldog-type” brachycephaly thus also affect the postcranial skeleton to a greater or lesser degree.

Lastly, short, broad crania in domestic dogs, i.e., the ones tending toward brachycephaly, have been found to be correlated with short and thick limb bones (Alpak, Mutuş and Onar 2004; Fischer and Lilje 2011; Smith et al. 2016), and also other skeletal elements exhibit peculiar shape changes that seem to be associated with the brachycephalic phenotype (Fig. 6). This may include changes to the pelvis and birth canal, which along with the oversized and “unnatural” shape of the brachycephalic head in domestic dogs and cats can cause dystocia due to fetal–pelvic disproportion, a condition that may require caesarean section (Bennett 1974; Gunn-Moore and Thrusfield 1995; Eneroth et al. 1999; Jackson 2004; Forsberg and Persson 2007; Evans and Adams 2010; Dobak et al. 2018).

## Genetic and developmental aspects of brachycephaly in domestication

Genetic and developmental studies have revealed that facial patterning is a complex process involving multiple gene regulatory networks, reciprocal signaling interactions, and hierarchical levels of control (Schneider 2018a). Although much insights have been gained in the last couple of years, there are still a lot of unknown factors, especially in domestic animals other than dogs. Apart from genetic factors, environmental and epigenetic factors may play a role as well in the generation of brachycephaly.

### Developmental basis of brachycephaly

While the size and shape of the face varies greatly across amniotes, at early embryonic stages the constituent parts all arise from comparable primordia, tissues, and cells (Schneider 2005; Young et al. 2014; Smith et al. 2015). The upper aspect of the face is derived from the frontonasal and paired maxillary primordia, while the lower portion forms from paired mandibular primordia. Neural crest mesenchyme (NCM) that migrates out of the midbrain and rostral hindbrain (i.e., rhombomeres 1 and 2) is the exclusive source of cartilage, bone, and other connective tissues within the facial primordia (Le Lièvre and Le Douarin 1975; Noden 1978; Couly, Coltey and Le Douarin 1993; Köntges and Lumsden 1996; Noden and Schneider 2006).

A broad range of experimental studies have identified many critical determinants that function during the induction, allocation, proliferation, and differentiation of NCM, and ultimately establish the size and shape of the face. Molecules such as Sonic Hedgehog (*SHH*), Fibroblast Growth Factors (*FGF*s), Wingless (*WNT*s), Transforming Growth Factor Beta (*TGFβ*), and Bone Morphogenetic Proteins (*BMP*s), which are primarily secreted from epithelial tissues that surround NCM in the facial primordia, have been implicated in affecting the shape and outgrowth of the jaw and facial skeletons especially by regulating skeletal polarity and axial growth (Schneider 2007; Fish and Schneider 2014b; Schneider 2015; Woronowicz and Schneider 2019). For instance, differential expression of *Bmp4* in NCM can generate variation in facial (i.e., beak) depth and width among birds including Darwin's finches, chicks, ducks, and cockatiels (Abzhanov et al. 2004; Wu et al. 2004; Wu et al. 2006) whereas jaw length appears to be regulated separately through other pathways (Abzhanov et al. 2006).

For its part, NCM controls the species-specific size and shape of the skeleton, as revealed through interspecific grafting experiments (Andres 1949; Wagner 1959; Noden 1983; Schneider and Helms 2003; Tucker and Lumsden 2004; Mitsiadis, Caton and Cobourne 2006; Noden and Schneider 2006; Lwigale and Schneider 2008; Fish and Schneider 2014a; Schneider 2018b). In particular, the use of a unique avian chimeric transplantation system that exploits species-specific differences between Japanese quail and white Pekin duck has revealed that NCM orchestrates the developmental programs underlying the size and shape of individual bones and cartilages within the facial skeleton (Schneider and Helms 2003; Eames and Schneider 2008). Chimeric “quck” embryos, which are duck hosts with quail donor cells, possess quail-like beaks, whereas chimeric “duail” exhibit duck-derived morphology in quail hosts. NCM accomplishes this complex task by controlling its own gene expression, cell cycle, and differentiation, as well as by regulating certain aspects of the developmental programs of adjacent host tissues including the pigmentation and patterning of epidermal appendages like feathers and the orientation and insertion sites of muscles (Eames and Schneider 2005; Tokita and Schneider 2009; Solem et al. 2011; Woronowicz et al. 2018; Schneider 2018a).

Initially, during the migration and allocation of NCM, quail and duck have distinct numbers of progenitors destined to form the jaw skeleton, with duck having significantly more cells (Fish et al. 2014). Then, as these populations expand, there is species-specific regulation of, and response to *SHH*, *FGF*, *BMP*, and *TGFβ* signaling in a species-specific manner, which likely modulates the proliferation, differentiation, and growth of skeletal progenitors, and generates variation in facial size and shape. Additionally, when these progenitors begin to differentiate into the cartilages and bones of the jaw and facial skeleton, they execute autonomous molecular and cellular programs for matrix deposition and resorption through patterns and processes that are intrinsic to each species (Eames and Schneider 2008; Merrill et al. 2008; Mitgutsch et al. 2011; Hall et al. 2014; Ealba et al. 2015). Thus, NCM-mediated changes to underlying developmental programs is likely to be a principal agent in the evolutionary foreshortening of the facial skeleton in brachycephaly. Correspondingly, impairing the migration of NCM has been found to be responsible for a brachycephalic phenotype in mice (e.g., Satokata and Maas 1994; Dixon et al. 2006; Noda, Nakamura and Komatsu 2015) and deficits in the amount of NCM that emigrates into the craniofacial primordia can cause neurocristopathies that produce widespread malformations to the jaws and face such as in the case of Treacher Collins syndrome (Kissel, André and Jacquier 1981; Jones et al. 2008).

During postnatal ontogeny, precocious ossification of cranial base synchondroses (i.e., the endochondral growth zones at the base of the cranium, which account for the longitudinal expansion of the cranium), in particular the spheno-occipital synchondrosis, has been found to be associated with “short headedness” and “bulldog-type” brachycephaly in domestic cattle (Julian et al. 1957), dogs (Stockard 1941; Schmidt et al. 2013), rabbits (Brown and Pearce 1945), and chicken (Landauer 1941). Similarly, but via genetic engineering, an interrelation between impaired endochondral ossification and aspects of “bulldog-type” brachycephaly has been shown in transgenic laboratory mice (Jolly and Moore 1975; Chen et al. 1999; Garofalo et al. 1999; Wang et al. 1999; Hajihosseini et al. 2001; Wadler Bloom et al. 2006) and rats (Pridans et al. 2018; Hume et al. 2020). In many of these varieties, the “bulldog-type” brachycephalic head shape is also associated with shorter legs, which grow in length via endochondral ossification of the growth plates at the apical ends of the long bones, analogous to longitudinal growth of the cranial base at the synchondroses. Indeed, many domestic dogs exhibiting “bulldog-type” brachycephaly, e.g., Pug and French Bulldog, also tend to have slightly curved, stout and short limb bones (Alpak et al. 2004; Smith et al. 2016) (Fig. 6). Impairment of endochondral ossification, i.e., chondrodysplasia or chondrodystrophy, as the developmental mechanism underlying “bulldog-type” brachycephaly is therefore a reasonable hypothesis. This hypothesis is also in accordance with the observation that the lower jaw in “bulldog-type” brachycephalic animals is longer than the upper jaw, creating the characteristic mandibular prognathism. The upper and the lower jaw of vertebrates have been shown to comprise different developmental modules (Klingenberg 1998), e.g., in dogs among domestic mammals (Stockard 1941; Curth, Fischer and Kupczik 2017), in which impairment of endochondral growth affects the upper jaw to a greater degree than the lower one due to the solely intramembranous ossification of the latter (Harvey 1985). The association of impairment of endochondral ossification with a bulging forehead and midface hypoplasia, along with a shortening of all limbs, is also known from humans (achondroplasia; Parrot 1878; Horton, Hall and Hecht 2007) and has been compared to “bulldog-type” brachycephaly in domestic dogs (e.g., Keith 1913; Stockard 1941; Marchant et al. 2017).

Many other cases of “bulldog-type” brachycephaly in domestic cattle and dogs show that the intertwined genetics make the relationships between impairment of endochondral ossification and cranial shape difficult to parse. Many chondrodysplastic domestic dog breeds do not overtly appear to be “bulldog-type” brachycephalic, but instead exhibit a mesocephalic cranial conformation (e.g., Basset Hound, Corgi, Dachshund). Similarly, the conspicuously “bulldog-type” brachycephalic Niata cattle have been found to exhibit normal-sized legs compared with non-brachycephalic cows and not a particularly early fusing cranial base synchondroses (Veitschegger et al. 2018). In contrast to this tendency toward “disproportionate dwarfism” characterizing “bulldog-type” brachycephalic forms to a greater or lesser degree and in a mosaic-like mode, “proportionate dwarfism” via a reduced level of growth hormones (Allan et al. 1978), a condition which is also known from humans, has been suggested to be the causative process underlying “allometric” brachycephaly (Stockard 1941; Schmidt et al. 2013).

Not only do cranial base synchondroses close earlier in “bulldog-type” brachycephalic dogs, but in adulthood, “bulldog-type” brachycephalic dogs exhibit more closing and closed cranial sutures than non-brachycephalic breeds (Geiger and Haussman 2016). Similarly, early closing cranial sutures associated with truncated faces have been found in brachycephalic cats (Schmidt et al. 2017) and genetically engineered mice (Hajihosseini et al. 2001). Such phenotypes in humans are known as a clinical symptoms indicative of various genetic diseases such as Crouzon, Apert, Muenke, Pfeiffer, and Saethre-Chotzen syndromes (Hajihosseini et al. 2001; Schmidt et al. 2017). However, underlying mechanisms and causality remain to be investigated. Similar examinations in the Niata cattle have been nonconclusive (Veitschegger et al. 2018).

Historically, the brachycephalic phenotype, particularly in domestic dogs, has been described as a retention of juvenile characters into adulthood, i.e., pedomorphosis (reviewed by Klatt 1913). This pedomorphic skull conformation typically includes a relatively short snout and a large braincase (Bolk 1926; Dechambre 1949; Wayne 1986; Morey 1992; Coppinger and Schneider 1995). Although “pedomorphic” skull proportions pertain to what is observed in small domestic dog breeds, i.e., cases of what is here described as “allometric” brachycephaly (Klatt 1913; Klingenberg 1998), the pedomorphosis hypothesis has been challenged and relativized on various grounds (Klatt 1913; Starck 1962; Rosenberg 1965; Drake 2011; Geiger et al. 2017). Even more so, the pedomorphosis hypothesis does not stand the comparison with “bulldog-type” brachycephaly: a “bulldog-type” skull conformation cannot be observed in any stage during the ancestral wolf ontogeny, although some general resemblances of skull structures, such as the short snout, may be prevalent (Klatt 1913; Drake 2011; Lord, Schneider and Coppinger 2016).

### Genetic basis of brachycephaly

Whether similar genetic mutations and developmental pathways are associated with “bulldog-type” brachycephaly in different domestic species and among different breeds/varieties remains unclear. Some authors argue that similar mutations are responsible among the different domestic dog breeds exhibiting “bulldog-type” brachycephaly (Bannasch et al. 2010). However, forms of chondrodysplasia associated with “bulldog-type” brachycephaly caused by a single genetic mutation may be lethal in homozygous individuals (e.g., cattle; Cavanagh et al. 2007). Nonlethal variants of brachycephaly, which might be fixed in certain breeds, however, seem associated with multiple, relatively mild genetic mutations (Schoenebeck and Ostrander 2013).

To date, progress toward understanding the genetics of canine brachycephaly has largely relied on genome-wide association studies (GWAS) to identify positional candidate genes. GWAS compare the allele frequencies of hundreds of thousands of DNA differences (“polymorphisms” or “genetic variants”) with respect to a phenotypic outcome such as face length. Polymorphisms whose allele frequencies segregate according to study subjects (i.e., brachycephalic vs. non-brachycephalic dogs) indicate regions of the genome that may determine head shape.

Implicitly, population studies including GWAS require DNA from large populations of unrelated animals. Given their popularity as pets, acquiring DNA from dogs is not particularly difficult. On the other hand, categorizing their head shapes is not straightforward. Bannasch et al. (2010) searched for genetic associations with head shape by comparing small brachycephalic dogs to large mesaticephalic/dolichocephalic pedigree dogs. In doing so, the authors identified a region on canine chromosome 1 that was associated with brachycephaly. Assuming that breed skull shapes are effectively standardized (i.e., all bulldog skulls appear the same, and, uniformly differ from all Great Dane skulls, which in themselves appear similar), subsequent GWAS used breed-averaged measurements and geometric morphometrics-derived ordination values from museum skull collections to serve as quantitative phenotypes to their respective genotyped populations (Boyko et al. 2010). These studies identified numerous additional genetic associations, notably those on chromosomes 1, 5, 26, 30, 32, and X. One of these, the association on chromosome 32, was fine mapped, which led to the identification of a putatively causative missense mutation in bone morphogenetic protein 3 (*BMP3*) (Schoenebeck et al. 2012). GWAS also helped to define the causal mutation of dog's disproportionate chondrodysplasia, an expressed retrogene insertion of fibroblast growth factor 4 (*FGF4*) (Parker et al. 2009). The *FGF4* retrogene insertion on chromosome 18 explains the short legs of “bulldog-type” and “allometric” brachycephalic breeds like the Pekingese, Pomeranian, Chihuahua, and Japanese chin. Subsequently, another *FGF4* retrogene insertion was identified on chromosome 12; this one insertion is carried by French bulldogs (Brown et al. 2017; Batcher et al. 2019).

The aforementioned GWAS, as well as others (Sutter et al. 2007; Vaysse et al. 2011; Rimbault et al. 2013; Hayward et al. 2016), were particularly effective at defining genetic variants associated with body size and by extension, “allometric” brachycephaly. Genes in proximity to polymorphisms with the most phenotypic explanatory power include insulin-like growth factor 1 (*IGF1*), insulin-like growth factor 1 receptor (*IGF1R*), high-mobility group AT-hook 2 (*HMGA2*), stanniocalcin 2 (*STC2*), growth hormone receptor (*GHR*), and SMAD family member 2 (*SMAD2*) and ligand-dependent nuclear receptor corepressor like (*LCORL*). Similar findings were made in rabbits (Carneiro et al. 2017).

Together, these studies began to reveal the “genetic tenets” of modern pedigree dog morphologies. First, many breed-defining traits such as leg length and body size are dictated by the consolidation of genetic variants that were inherited prior to, or during, breed formation. Therefore, Dachshunds and Pekingese must share ancestry because both breeds’ short legs are caused by the *FGF4* retrogene insertion on chromosome 18. (Convergence can be excluded in this case, since the same causative mutation and surrounding haplotype are fixed in both breeds. However, short-leggedness in some other breeds is caused via a convergent mechanism; e.g., Parker et al. 2009; Brown et al. 2017). Similarly, body size reduction as it occurs in toy, small-, and medium-sized dogs appears to rely on the sum total contributions of a small number of genetic variants that presumably influence *IGF1*, *IGF1R*, *HMGA2*, *STC*, and *SMAD2* protein production or function. A second related tenet of modern pedigree dog morphologies is that few genetic variants appear to explain traits like leg length and body size and their phenotypic effect sizes are quite large (Boyko et al. 2010; Rimbault et al. 2013; Hayward et al. 2016). This makes sense when we reflect on how such unusual morphologies were propagated in the first place: variants with large effect sizes manifest traits that are visually recognizable, as is required to guide selective breeding.

In terms of studying body size, canine geneticists have largely ignored individual-level morphometrics and instead have relied on increases in study population size and/or genotype density to identify additional genetic associations (Hayward et al. 2016; Mansour et al. 2018; Plassais et al. 2019). As a consequence, even more associations with morphological traits have been described, but their interpretation is confounded by the inability to separate morphological effects of variants on sub-anatomy and allometry. Moreover, reliance of pedigree dogs in these GWAS risks false positive associations that emerge because of shared ancestry that is “coincidental” to morphological traits. Finally, the assumption that breed-defining traits are always fixed within breeds is rarely absolute, especially for complex morphology like skull form whose underpinnings are polygenic.

In an attempt to avoid these pitfalls, Schoenebeck and colleagues pioneered the use of veterinary clinical imaging data to provide individually coupled genotypes and morphometrics. Pedigree and mixed breed ancestries were studied to identify genetic associations with cranial size and size-corrected (non-allometric) face shape (Marchant et al. 2017). Associations with cranial size independently validated the positional candidate genes *IGF1*, *HMGA2*, *SMAD2*, *LCORL* and the *FGF4* retrogene (chromosome 18). Although the authors assumed a common growth trajectory across study subjects, analysis of face length reproduced the association of chromosome 1 that was previously reported by Bannasch et al. (2010). Fine mapping of the locus, combined with whole-genome sequence analysis, revealed a LINE-1 retransposon within an intron of the gene SPARC related modular calcium binding 2 (*SMOC2*) gene. Nearly all tested “bulldog-type” brachycephalic dogs were fixed for this LINE insertion. Others carried the LINE-1, including Staffordshire Terriers, Pitbulls and toy dogs, including the “allometric” brachycephalic Chihuahua. Critically, the individualized data demonstrated a semidominant effect, as a single copy of the LINE was associated with an intermediate reduction in face length. Moreover, insertion of the LINE-1 was associated with a reduction in *SMOC2* transcription and missplicing of its transcripts.

The LINE-1 insertion at *SMOC2* is likely to be a major determinant of face length, but yet it is clear that even among morphometric analyses of “bulldog-type” brachycephalic dogs, such dogs differ among themselves in terms of relative face length, palate angulation, and more. Some of these differences are undoubtedly due to allometry, thus the influences of genetic variants of the *IGF1*, *HMGA2*, and other cranial scale loci are relevant. *BMP3* variation is associated with face length, but only among small through medium breed dogs. Using whole-genome sequencing, a deletion in the dishevelled 2 (*DVL2*) gene was identified among bulldog breeds with a screw tail, a breed-defining condition where the caudal-most vertebrae in the tail fail to form, and the remaining tail vertebrae are malformed (Mansour et al. 2018). *DISHEVELLED* proteins help modulate *WNT* signaling and family members *DVL1* and *DVL3* are recognized to play critical roles in development including anteroposterior growth (“convergent extension”) and promotion of osteogenesis (Day et al. 2005). Although far less is known about *DVL2* protein's function, it is reasonable to speculate that the deletion mutation, which truncates the protein's C-terminus and reduces *DVL2* phosphorylation, alters axis formation and/or osteogenesis. Future studies that investigate the morphological differences between mixed breed dogs are required to assess the morphological effects imparted by the *DVL2* mutation in the absence of other cranium-associated genetic variants. Moreover, future dog studies with deep population sampling will be required to explore and even adjust for the growth trajectories of individual subjects using genotypic information.

## Possible reasons for the prevalence or lack of brachycephaly in domesticated species

Brachycephalic varieties/breeds are known from different domesticated species and the number of such breeds per species varies (see earlier, Table 2; Fig. 1). However, in other species, no such breeds are known and incidences of an overshot lower jaw, i.e., “spontaneous” brachycephaly, are usually regarded as pathologies (e.g., in horses) (Fig. 1). Therefore, the question remains as to why some species show no varieties or breeds characterized by brachycephaly, whereas other species do.

### Possible reasons for a lack of brachycephalic varieties in some domesticates

Reasons for a lack of brachycephalic varieties/breeds in certain domesticates may theoretically and generally include (1) a lack of genetic and/or phenotypic variation that could be selected in the first place, (2) a strong selection against the phenotype, either naturally or artificially, or (3) the absence of artificial selection in favor of the trait. Since the first point—“occasional” brachycephalic varieties—has been reported for most domesticates (see earlier), we speculate that a potential lack of variation might not play a major role in the lack of brachycephalic varieties/breeds in some domestic species. In contrast, the second and third points may be more important in explaining the observed pattern (Fig. 1).

**Fig. 7:**
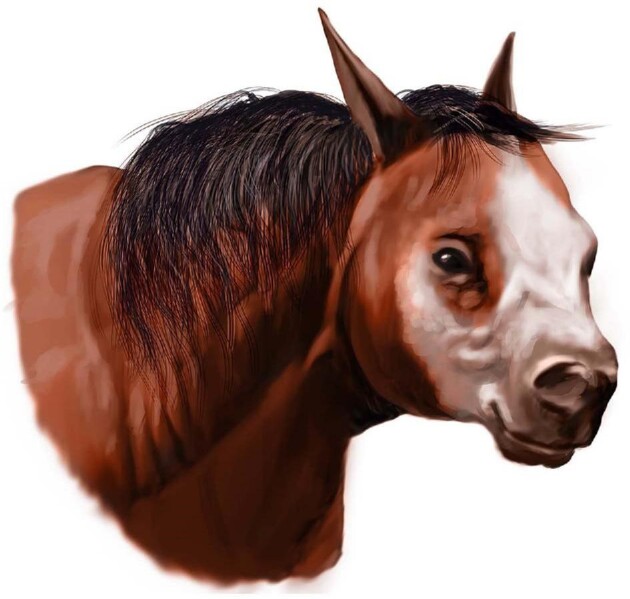
Example of an imaginary variety, the brachycephalic domestic horse. In some domestic species, such as the horse as depicted here, brachycephalic varieties/breeds are not known and might even be perceived grotesque. Artwork by Jaime Chirinos.

Stockard (1941, 20) speculated that humans would only be interested in artificially selecting, for instance, horses for traits that are of benefit to them, such as riding and working, and not to preserve “the odd, grotesque, or useless” (Fig. 7). According to this line of argumentation, one would suspect that there are fewer brachycephalic varieties in domestic species that are of substantial economic value to humans, as livestock or working animals. However, aesthetic views may vary in different human societies and cultures (Epstein 1971) and this might not be a universal rule. For horses, it has been suggested that the lack of brachycephalic varieties—and other “signs of degeneration”—is not to be found due to the need for them being “constitutionally hardy” (Adametz 1926, 95; loosely translated from German).

Strong natural selection against a brachycephalic phenotype might be related to the various health issues associated with brachycephaly (as described earlier), which—if untreated—may be lethal or reducing the fitness of affected individuals. Further, natural selection against a brachycephalic phenotype might stem from the mode of growth of the teeth in some species, specifically high crowned or ever-growing teeth. In equids and ruminants, permanent cheek teeth continue growing in length for a couple of years after occlusal contact is established and before the roots close and growth ceases (hypsodonty) (Habermehl 1975; Harvey 1985; Hillson 2005; Ungar 2010). In addition to the check teeth, the incisors continue growing after eruption into occlusion in horses (Sisson and Grossman 1953). In rodents and lagomorphs, the incisors are ever-growing (hypselodont) and in caviid rodents (here the guinea pig) and leporid lagomorphs (here the rabbit), also the cheek teeth are hypselodont (Ungar 2010).

In domestic equids, there are occasional occurrences of individuals exhibiting cranial morphology reminiscent of brachycephaly (Harvey 1985) but this is never a breed defining characteristic. In horses, a shortening of the maxilla and therefore mandibular prognathism (termed “monkey mouth” or “sow mouth”) results in dental malocclusion and may lead to overgrowth of the opposing arcade, which impairs mastication (Harvey 1985). Such malocclusions have been described to be the result of breeding practices for desired head shapes and are particularly prevalent in ponies and miniature horses (Wiggs and Lobprise 1997; Heck, Sánchez-Villagra and Stange 2019). Similarly, the most common dental problem in lagomorphs and rodents is malocclusion of the teeth (Studer 1975; Wiggs and Lobprise 1997; Müller et al. 2014, 2015; Böhmer and Böhmer 2017). Such malocclusion may lead to overgrowth of the ever-growing incisors and cheek teeth, which may be fatal if there is no timely medical intervention (Studer 1975; Wiggs and Lobprise 1997; Crossley 2003; Reiter 2008). However, if there is sufficient medical care, such phenotypes might survive if desired (British Veterinary Association 2017).

Even if the proportion of the upper and the lower jaw is not changed in any of the horse breeds, “allometric” brachycephaly has so far not been observed in domestic equids either (Heck et al. 2019). Specifically, even the smallest horse breeds (e.g., Falabella, Shetland pony) do not exhibit a relatively shorter face than larger horse breeds (e.g., English Thoroughbred, Shire). A similar pattern has been found in grass-feeding bovids and equids and termed the “long-face hypothesis” (Spencer 1995; Cardini 2019; Heck et al. 2019). This pattern might be related to the need to maintain feeding efficiency, independent of body size. This is especially important in regard of the relatively energy poor grass diet. Possibly as a consequence of such constraints, miniature horses have similar-sized molar teeth compared with larger horses, which is often leading to dental health issues in these small breeds (Wilson 2012).

In summary, we suggest that relatively strong natural selection may be acting against the brachycephalic phenotype in various species due to brachycephaly-related morbidity and mortality and/or due to the presence of high-crowned or ever-growing teeth and the potentially fatal risk of dental malocclusion in particular species. These constraints may be combined with a lack of human interest in maintaining brachycephalic lines when they arise due to a lack of obvious benefits (i.e., economic) associated with the phenotype. However, there are a few goat breeds and the Niata cattle in which brachycephaly is a breed-defining characteristic (Table 2). This suggests that such constraints may be overcome in livestock (see later), either via random processes due to a small founder population, as has been suggested for the Niata cattle (Veitschegger et al. 2018), or via selection (see next section). To test this, more studies on dental health in ungulates and rodents with brachygnathia superior as well as a more profound knowledge on aesthetic perception and ceremonial or symbolic culture across human cultures would be crucial.

Also in clades other than ungulates, rodents and lagomorphs, extreme brachycephaly is associated with morbidity, e.g., in cats and dogs (see earlier; e.g., Waters 2017; Bessant et al. 2018). Human intervention and medical care are often required for animals exhibiting extreme brachycephaly in order to mitigate the pathologies, at least for companion animals in industrialized countries (e.g., Riecks, Birchard and Stephens 2007). Additionally, the popularity of the brachycephalic phenotype as a cultural phenomenon and when health problems become normalized (Packer, Hendricks and Burn 2012) lead to the artificial maintenance of the phenotype, despite its indisputable disadvantages for individual fitness (Thomson 1996). (There are also endeavors to breed less extreme forms, e.g., Continental and Old English Bulldog [Krämer 2009], but changing entire breeds to a healthier state is a long-term process [Ravn-Mølby et al. 2019]). Populations of dogs and cats that are free ranging and exhibiting different degrees of socialization with humans since generations, e.g., village and feral (i.e., wild domestic) dogs and cats, do not usually exhibit brachycephaly to a pathological degree.

### Possible reasons for a prevalence of brachycephalic varieties in some domesticates

As opposed to the reasons why in some lineages brachycephaly is rare or does not occur, reasons for the prevalence of brachycephalic varieties/breeds in certain domesticated species may include (1) the prevalence of such genetic and/or phenotypic variation that can be selected, including small founder populations in which such varieties arise due to genetic drift, e.g., in the Niata cattle (Veitschegger et al. 2018), (2) lack of selection against the phenotype (e.g., bunodonty, instead of hypsodonty, in cats, dogs, and pigs; see earlier), and (3) natural and/or artificial selection in favor of the phenotype.

Specific characteristics of domestic varieties and breeds may not only be the result of artificial selection for these traits but may also occur due to an adaptation to particular environmental conditions. Such varieties, which are usually geographically restricted, are termed “landraces” and have historically been common in various species of farm animals as well as dogs. As brachycephaly might not constitute a pathology in all cases, adaptations might be the cause of a brachycephalic phenotype in some domestic varieties/breeds. The strongly convex nose in Jamnapari goats, which may result in an overshot lower jaw (Figs. 1 and 3; Table 2), in conjunction with the long ears (Fig. 3), has been speculated to lead to a preference for browsing, rather than grazing, in these animals (Rout et al. 2002). Rout et al. (2002) argue that, if the lower jaw protrudes beyond the upper jaw and if the long ears touch the ground before the mouth, leading to partial blindness as the head is lowered, browsing leaves from brushes is easier than grazing from the ground. Although the brachycephalic head conformation has been suggested to leave these goats practically starving when there is only grass available (Rout et al. 2002), brachycephaly may constitute a specialization for browsing and therefore adaptation to a particular environment, favored by natural as well as artificial selection. (Environmental conditions and vegetation across the geographical distribution of brachycephalic goats are not uniform, but many breeds originated in semiarid climate zones [Table 2].) In contrast to goats, which are also feeding on shrubs, sheep are more relying on grasses as their diet (Castelló 2016) and this may explain the lack of fixed brachycephaly in any variety/breed of this domestic species.

Contrary to the hypothesis put forward for brachycephalic goats earlier, the overshot lower jaw in combination with airorhynchy and the subsequent inability of the upper and lower lips to meet in the extinct Niata cattle (Figs. 1 and 3) have been speculated to have been a disadvantage during droughts, as these animals were incapable of browsing (Darwin 1878) or to graze on dry or low standing grasses that cannot be ingested with the help of the tongue (Adametz 1926). This may speak against a specific adaptation of head shape in these animals and the fixed brachycephalic head conformation might be the result of genetic drift (Veitschegger et al. 2018). On the other hand, finite element analysis in Niata cows compared with more “wild-type” cattle has shown that the brachycephalic and airorhynch skulls exhibit lower magnitudes of stress during biting (Veitschegger et al. 2018), which may suggest some adaptive advantage of these cows compared with other breeds, e.g., to process tough food. Further, the brachycephalic head conformation might have hampered these animals’ ability to feed to such a degree that they were particularly dependent on human provisioning of food and thus affected animals were less likely to roam, instead were more likely to stay with their human keepers. Characteristics that render herd managing easier are likely to be particularly valuable and might therefore artificially be selected for, as is known for the short leggedness in Ancon sheep (Landauer and Chang 1949). These opposing views regarding the ability to graze and browse in brachycephalic ruminants with overshot lower jaws are highly speculative and would require further studies on feeding behavior of brachycephalic breeds (albeit not possible in the extinct Niata cattle).

Similarly, airorhynchy in some pig breeds has been speculated to be associated with selection for a more efficient exploitation of food supplies in (certain) domestic settings: airorhynchy generally leads to an elevation of the temporomandibular joint in relation to the occlusal plane of the teeth (Fig. 1). This elevation makes possible the more even distribution of bite forces across the teeth and improvement of the mechanical efficiency of the masticatory muscles (Thenius 1970). Further, the more laterally protruding canines in these pigs enable the jaws to also move horizontally (Thenius 1970). Similar specializations as in these brachycephalic pigs are also found in wild species with more herbivorous diets than their relatives, e.g., orangutans compared with African great apes (Shea 1985; Neaux et al. 2015). Anecdotally, short snouted Middle White and Kunekune pigs are said to root/dig less than other breeds, which may be considered a favorable characteristic in animal husbandry and a reason to artificially select these traits in these breeds. However, also here, such considerations remain speculative.

In domestic dogs, potential reasons for favoring and artificially selecting a brachycephalic phenotype seem more straightforward. The tendency toward (i.e., relatively mildly expressed) “bulldog-type” brachycephaly may constitute an artificially selected trait in dogs, which purportedly have been bred for bullfighting and for holding down prey during a hunt of for slaughter (Räber 1993; Thomson 1996; Krämer 2013). The short snout and more laterally and inferiorly displaced zygomatic arches as well as dorsoventrally higher cranial vault allow for larger temporalis muscles, which may increase bite force (Ellis et al. 2009; Selba et al. 2019). Brachycephaly in domestic dogs is usually also associated with relatively stout limbs (Fig. 6), round chest, and the tendency to bite and not let go, which may also be regarded as adaptations for fighting. Specifically, individuals in a fighting-dog breeding line that are less well suited for fighting than other individuals from the same line will likely have a reduced fitness because they will be less likely to be bred or they will even be killed (Thomson 1996; Alpak et al. 2004; Fischer and Lilje 2011; Smith et al. 2016). Taken together, these points would point to positive artificial selection for brachycephaly in some (previously) working or sporting dogs (e.g., Bulldogs). However, in the aftermath of the prohibition of these practices, present-day cases of extreme brachycephaly in some breeds might be the result of aesthetic considerations and corresponding breeding practices (or accident) (Wilcox and Walkowicz 1989; Räber 1993; Thomson 1996).

In brachycephalic toy dogs, rabbits, and cats, human preference for a child-like appearance, i.e., round face, large eyes, and small nose, may have led to selection for brachycephalic forms (Harvey et al. 2019). However, at least in cats, extreme brachycephaly seems to be less preferred than more mildly brachycephalic or non-brachycephalic head conformations (Farnworth et al. 2018). In general, child-like features may have provoked protective instincts in humans and might be the reason why such phenotypes have been selected for in the first place (Fournier 2002). Finally, in some cases, the formation of a brachycephalic breed, independent of the taxon, might be based on individual human preferences for the “exotic” (Sykes 2014).

## Conclusions

This review highlights the complex nature of “brachycephaly,” discusses associated morphological traits, especially diseases, and outlines the current state of knowledge about potential genetic and developmental mechanisms, as well as hypothesis about the patterns that we see today in terms of the occurrence or lack of this skull shape in the different domestic species. The main points are stated in the following:

1. “Brachycephaly” is a term that broadly describes shortness of the head, including the snout in animals. The shortness of the head, however, is a superficial description with variable morphological, genetic, and developmental underpinnings.
2. Brachycephalic varieties and breeds are known from various domestic species, including cats, dogs, rabbits, cattle, goats, and pigs among mammals; some pigeons and chicken varieties among birds; and some fishes. However, in some other domestic species, brachycephalic varieties/breeds are lacking (e.g., horses and sheep; note that occasionally, brachycephalic individuals might occur in these species, but that they are usually regarded pathological).
3. In general, three main morphotypes of brachycephaly can be discerned: “bulldog type” brachycephaly (including an upward tilted snout, i.e., airorhynchy), “katantognathic” brachycephaly (including a downward tilted anterior part of the snout, i.e., katantognathy), and “allometric” brachycephaly (resulting in a short snout length in small varieties/breeds due to allometric scaling).
4. Genetic and developmental underpinnings of brachycephaly are complex, involving multiple gene regulatory networks, reciprocal signaling interactions, and hierarchical levels of control. Although much is still unknown, especially in animals other than dogs and cats, much insight into genetic variants and developmental mechanisms underlying the different types of brachycephaly could be gained in recent years.
5. Extreme cases of brachycephaly are associated with a large range of pathologies, affecting all stages of life history and different organ systems. Thus, substantial animal welfare issues are associated with the breeding of extreme brachycephalic forms, and ethical considerations warrant a discussion about adjustments of breeding standards and interpretation thereof.
6. Reasons why brachycephalic varieties/breeds are not found in some domestic species might be the result of biological factors (purifying selection) as well as cultural factors, including lack of artificial selection for the phenotype.

A better understanding of these abovementioned aspects of brachycephaly bears great potential for various fields of research, including domestication research, veterinary and human medicine, and developmental and evolutionary biology, and might also be pivotal in more applied fields, such as animal breeding and welfare, with the potential to mitigating suffering in extreme cases of brachycephaly. Especially, further insights into behavioral associations, as well as genetic and developmental underpinnings of the different types of brachycephaly in different animal species, are crucial for a better understanding of this peculiar phenotype. This is particularly the case in species that are less well studied than domestic dogs and cats. Ultimately, deciphering the cause and effect of putatively causal variants on skull morphology will require vastly larger study populations with individualized phenotypes and genotypes.

## Supplementary Material

obab023_GeigerEtAl_Brachycephaly_Appendix
