# Erratum

**DOI:** 10.1093/iob/obab031

**Published:** 2021-10-25

## Abstract

Erratum to: Exceptional changes in skeletal anatomy under domestication: the case of brachycephaly

*Integrative Organismal Biology*, Volume 3, Issue 1, 2021, obab023, https://doi.org/10.1093/iob/obab023

Due to production error in the above article, figure 1 has been updated as follows online:

**Fig. 1:**
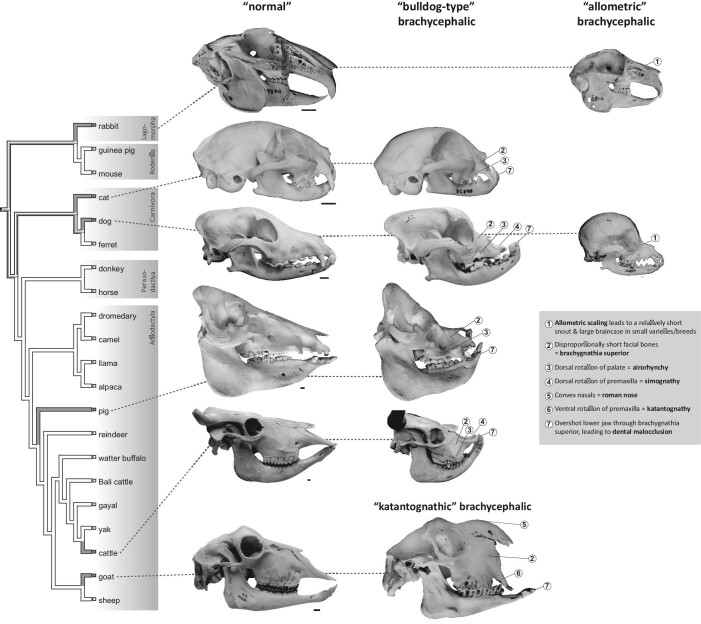
Summary of brachycephalic varieties in domestic mammal species. Cladogram (branches contain no information on divergence times) shows ancient mammal domesticates (domesticated >500 YBP, see text; tree topology is according to Meredith et al. 2011 and Agnarsson and May-Collado 2008). Gray branches indicate species with at least one variety/breed where a brachycephalic phenotype is considered to occur relatively consistently or is breed defining and not just occurring occasionally, e.g., as a pathology (see text and Table 2). Skulls categorized as “normal” (left column) represent the non-brachycephalic condition in the respective domesticates. Skulls in the other columns represent brachycephalic varieties/breeds, according to the groupings as described in the text (“bulldog type,” “katantognathic,” and “allometric”). Numbers indicate discussed characteristics of the brachycephalic phenotype. It is evident that not all domestic species are represented by brachycephalic varieties and that the phenotype that is usually termed “brachycephalic” is variable in the different species. From left to right and top to bottom: Angora rabbit (Zoologisches Institut/Populationsgenetik [former Institut für Haustierkunde], Christian-Albrechts-Universität zu Kiel, Germany; I.f.H. 6489, mirrored); Polish rabbit (“Hermelinkaninchen,” I.f.H. 5348); domestic cat of unknown breed (I.f.H. 12689); Persian cat (I.f.H. 20428, mirrored); domestic dog of unknown breed (Paleontological Institute and Museum, University of Zurich; PIMUZ A/V 608); Boxer (PIMUZ A/V 2836, mirrored); Chihuahua (Albert Heim collection at the Naturhistorisches Museum Bern, Switzerland; NMBE 1052001); domestic pig of unknown breed (Zoological Museum, University of Zurich; ZMZH 17676); brachycephalic domestic pig of unknown breed (Nehring-Collection [Zoologische Sammlung der Königlichen Landwirtschaftlichen Hochschule zu Berlin] at the Museum für Naturkunde Berlin, Germany; ZMB_Mam_106884); domestic cattle of unknown breed (PIMUZ A/V 2, mirrored); Niata cattle (Natural History Museum of Denmark; NHMD-ZMK-MK-1109, mirrored; courtesy Kristian Murphy Gregersen); mixed breed goat (Center of Natural History, University of Hamburg; ZMH 10895, mirrored); and “Egyptian goat” (“Ägyptische Ziege”; Naturmuseum Wien, Austria; NMW 2074). “Normal” skulls are scaled to the same length across species and brachycephalic skulls are scaled to the non-brachycephalic ones of the same species; scale bars equal 1 cm. Specimens are dentally mature, except the brachycephalic pig. Cattles are shown with (graphically) cut horns. Erratum concerning figure 1e in Veitschegger et al. (2018): the schematic depiction of a brachycephalic cat skull (modified from Schlueter et al. 2009) shows a Persian cat, not a Siamese cat.

