# Supplementary material for: Exceptional Changes in Skeletal Anatomy under Domestication: The Case of Brachycephaly
Source: Integr Org Biol. 2021 Aug 16;3(1):obab023. doi: 10.1093/iob/obab023 (PMC8366567; doi:10.1093/iob/obab023)
Supplement: obab023_GeigerEtAl_Brachycephaly_Appendix [file obab023_geigeretal_brachycephaly_appendix.docx]

*Review Article Appendix – Integrative Organismal Biology: A Journal of the Society for Integrative and Comparative Biology*

**Exceptional changes in skeletal anatomy under domestication: the case of brachycephaly**

M. Geiger^1^*, J. J. Schoenebeck^2^, R. A. Schneider^3^, M. Schmidt^4^, M. S. Fischer^5^, M. R. Sánchez-Villagra^1^

Supplementary Table S1. Measurements of the prebasial angle in Chihuahua skulls housed in the Albert-Heim-Collection at the Naturhistorisches Museum Bern (NMBE), Switzerland, as described by Nussbaumer (1982) and Baxter and Nussbaumer (2009)

| **Collection ID** | **Breed** | **Sex** | **Age[years]** | **Year of collection** | **Prebasial angle[°]** |
| --- | --- | --- | --- | --- | --- |
| NMBE1051989 | Chihuahua | f | 1.4 | 1987 | 164.5 |
| NMBE1051992 | Chihuahua | f | 2.06 | 1987 | 154.5 |
| NMBE1051998 | Chihuahua | f | 7.8 | 1992 | 169 |
| NMBE1052001 | Chihuahua | m | 11.2 | 1994 | 164.5 |
| NMBE1052003 | Chihuahua | f | 2.08 | 1988 | 164.5 |
| NMBE1052004 | Chihuahua | f | 4.9 | 1990 | 169 |
| NMBE1052006 | Chihuahua | m | na | 1996 | 168.5 |
| NMBE1052008 | Chihuahua | m | na | 1996 | 163.5 |
| NMBE1052009 | Chihuahua | f | 10.7 | 1999 | 174 |
| NMBE1052346 | Chihuahua | f | 5.3 | 1999 | 168 |
| NMBE1052347 | Chihuahua | f | 13.2 | 1999 | 175.5 |
| NMBE1052353 | Chihuahua | f | 3.8 | 1996 | 164.5 |
| NMBE1053116 | Chihuahua | f | 13.1 | 2000 | 169 |
| NMBE1053117 | Chihuahua | f | 12 | 2001 | 174 |
| NMBE1053118 | Chihuahua | f | 15.3 | 2002 | 173 |
| NMBE1055719 | Chihuahua | m | 12.4 | 2005 | 170 |
| NMBE1058267 | Chihuahua | f | 12.9 | 2003 | 170 |
| NMBE1059986 | Chihuahua | m | 9.5 | 2009 | 175 |
| NMBE1060875 | Chihuahua | f | 4.1 | 2008 | 169 |
|  |  |  |  | Breed mean prebasial angle [°] | 168.42 |
|  |  |  |  | Standard Deviation | 4.93 |

**Supplementary references**

Baxter, I.L., Nussbaumer, M., 2009. Evidence of morphometric variation in an Iron age dog cranium from Trumpington, Cambridgeshire, UK. Archaeofauna 18, 67-76.

Nussbaumer, M., 1982. On the variability of dorso-basal curvatures in skulls of domestic dogs. Zool Anz 209, 1-32.
